# The Effects of Both Recent and Long-Term Selection and Genetic Drift Are Readily Evident in North American Barley Breeding Populations

**DOI:** 10.1534/g3.115.024349

**Published:** 2015-12-29

**Authors:** Ana M. Poets, Mohsen Mohammadi, Kiran Seth, Hongyun Wang, Thomas J. Y. Kono, Zhou Fang, Gary J. Muehlbauer, Kevin P. Smith, Peter L. Morrell

**Affiliations:** * Department of Agronomy and Plant Genetics, University of Minnesota, Saint Paul, Minnesota 55108; † DuPont Pioneer, Mankato, Minnesota 56001

**Keywords:** introgression, breeding, evolution of crops, genetics, identity by state

## Abstract

Barley was introduced to North America ∼400 yr ago but adaptation to modern production environments is more recent. Comparisons of allele frequencies among growth habits and spike (inflorescence) types in North America indicate that significant genetic differentiation has accumulated in a relatively short evolutionary time span. Allele frequency differentiation is greatest among barley with two-row *vs.* six-row spikes, followed by spring *vs.* winter growth habit. Large changes in allele frequency among breeding programs suggest a major contribution of genetic drift and linked selection on genetic variation. Despite this, comparisons of 3613 modern North American cultivated barley breeding lines that differ for spike-type and growth habit permit the discovery of 142 single nucleotide polymorphism (SNP) outliers putatively linked to targets of selection. For example, SNPs within the *Cbf4*, *Ppd-H1*, and *Vrn-H1* loci, which have previously been associated with agronomically adaptive phenotypes, are identified as outliers. Analysis of extended haplotype sharing identifies genomic regions shared within and among breeding populations, suggestive of a number of genomic regions subject to recent selection. Finally, we are able to identify recent bouts of gene flow between breeding populations that could point to the sharing of agronomically adaptive variation. These results are supported by pedigrees and breeders’ understanding of germplasm sharing.

The evolution of breeding populations encompasses processes that reasonably mimic the evolution of natural populations, but in accelerated time (Ross-Ibarra *et al.* 2007). Comparative population genetic approaches can be used for the identification of genes underlying adaptive variation and to understand the effects of demographic patterns on diversity without specific phenotypic information (Nielsen *et al.* 2007; Ross-Ibarra *et al.* 2007).

Breeding populations are subject to demographic effects, such as founder events associated with their initiation (Martin *et al.* 1991), and potentially bottlenecks, and gene flow from other breeding populations or exotic sources. Although the primary goal of breeding programs is the selection of progeny with improved agronomic adaptation, these demographic effects also contribute to differences in allele frequency among breeding populations. Thus the differences among populations are a combination of genetic drift and selection for both agronomic traits and for improved local adaptation. This selection also involves some degree of concomitant linked selection. Understanding the selection and demographic history, including the effects of genetic drift and migration in breeding populations, can accelerate crop improvement (Ross-Ibarra *et al.* 2007), for example through the identification of loci involved in domestication and improvement (*e.g.*, Cavanagh *et al.* 2013; van Heerwaarden *et al.* 2012; Hufford *et al.* 2012), identification of introgression between domesticates and wild relatives (*e.g.*, Hufford *et al.* 2013), and the determination of specific donor individuals contributing to disease resistance variants (*e.g.*, Fang *et al.* 2013).

The history of population differentiation due to selection can be investigated by the identification of loci with large differences in allele frequency (Cavalli-Sforza 1966). Selection at different timescales can be detected by complementary techniques. For older and longer-term selective events we can use fixation indices or *F*-statistics (Nielsen *et al.* 2007) to measure allele frequency differences between populations. Variants with large differences in allele frequency among subpopulations, as measured by *F*_ST_, may have been subject to selection (Lewontin and Krakauer 1973). A high degree of haplotype sharing between individuals can be indicative of more recent selection (Pritchard *et al.* 2010; Horton *et al.* 2012). Haplotype sharing based analyses can identify specific haplotypes (and their underlying sequence variants) subject to selection based on their relative frequencies in a population (Hudson *et al.* 1994; Innan *et al.* 2005).

Migration between populations can contribute to adaptive variation (Slatkin 1987). Identity by state (IBS) analysis is a sensitive approach for the identification of specific genomic regions involved in migration between distinct populations. IBS analyses have been used in studies of barley and maize to identify specific genomic regions involved in adaptation to new environments (Hufford *et al.* 2013) and disease resistance genes derived from exotic germplasm (Fang *et al.* 2013).

In the present study we use SNP genotype data from the Barley Coordinated Agricultural Project to investigate the breeding history of North American barley breeding populations. Barley (*Hordeum vulgare* subsp. *vulgare*) was introduced to North America by European colonists as early as 1602 as a crop essential for beer production (Weaver 1950). Early successes in barley production in North America involved introduction of cultivars adapted to similar environmental conditions. In the eastern growing region of the United States barley was introduced from northern Europe, including English malting varieties; and in the western growing region from Mediterranean feed cultivars (Weaver 1943). Notable exceptions from outside northern Europe included “Manchuria” from northeastern China, which was well adapted to the Upper Midwest growing environment, “Stavropol” from Russia, which was the most important cultivar in the Lower Midwest (especially Kansas), and “Trebi” from the southern shores of the Black Sea in Turkey, which was found to be particularly productive in western regions under irrigated conditions (Weaver 1943; Wiebe and Reid 1961). Despite the use of germplasm from multiple Old World sources, the set of founder cultivars was relatively narrow, which likely contributed to reduced diversity in modern North American cultivars (Martin *et al.* 1991).

Barley production is frequently divided into spring and winter growth habit. Spring barley dominates in North America, while winter barley is grown in more southerly latitudes and moderate coastal climates (Weaver 1950). Spring and winter barley have been bred separately by breeding programs located in distinct geographic regions. Further trait requirements by end-use markets (*i.e.*, malting, feed, and food) and the establishment of breeding programs for two- and six-rowed spike-type contribute to highly structured barley populations (Cuesta-Marcos *et al.* 2010; Hamblin *et al.* 2010; Wang *et al.* 2012; Zhou *et al.* 2012).

Our analysis focuses on four major questions. First, which of the factors, including breeding populations, growth habit, and spike-type, contribute most directly to genetic differentiation among our samples? Second, to what extent are loci identified as major contributors to phenotypic variance in Old World barley germplasm contributing to allele frequency differences in North American breeding populations? Third, can we identify evidence of recent or long-term selection acting on breeding populations and what loci are involved? Fourth, how have patterns of shared ancestry and migration contributed to diversity and relatedness in current breeding populations?

Allele frequency-based analyses of the North American breeding programs indicate a large contribution of genetic drift and linked selection to genetic variation. Patterns of allele frequencies observed in loci known to be involved in adaptive traits in the Old World are recapitulated in North American breeding programs. Additionally we were able to identify populations and specific genomic segments involved in more recent gene flow.

## Materials and Methods

### Plant materials

Genotypic data from a total of 3971 barley accessions including cultivars, advanced lines, and genetic stocks from barley breeding programs that participated in the Barley Coordinated Agricultural Project (CAP) were downloaded from The Triticeae Toolbox (T3) (http://triticeaetoolbox.org/). Barley samples are representatives of 4 yr of germplasm enhancement (2006–2009) from 10 breeding programs. These breeding programs include most barley growing regions and market end-uses in the United States. For more information about the programs see Hamblin *et al.* (2010) and Wang *et al.* (2012).

**Figure 1:**
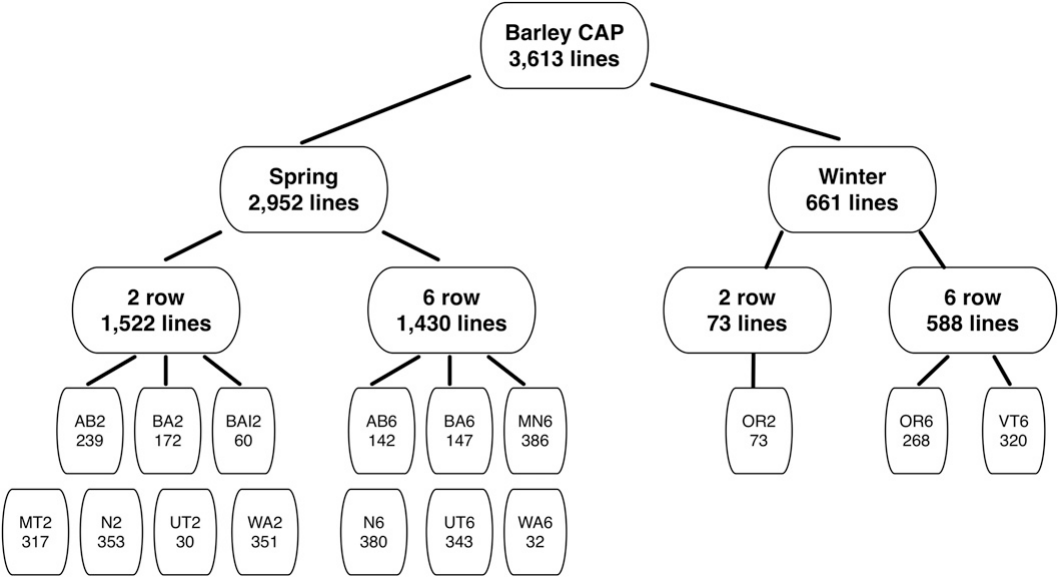
Breeding programs. University of Idaho in Aberdeen (AB), Busch Agricultural Resources, Inc. (BA), Busch Agricultural Resources, Inc. International lines (BAI), Oregon State University (OR), Utah State University (UT), Washington State University (WA), Montana State University (MT), Virginia Polytechnic Institute and State University (VT), North Dakota State University two-rowed (N2), North Dakota State University six-rowed (N6), and University of Minnesota (MN).

The breeding of two- and six-rowed barley within a single program often involves different objectives; thus we consider these types as independent populations resulting in 16 breeding populations (see Table 1). Hereafter, the following notation was used when referring to each breeding population: “breeding program’s name abbreviation” followed by 2 or 6 for two- and six-rowed spikes, respectively, as described in Table 1. The Busch Agricultural Resources international program referred to here as BAI2, was separated from the North American two-rowed lines, referred to here as BA2. There were 10 six-rowed accessions from Oregon mislabeled in T3 as two-rowed (P. Hayes, personal communication); we used the corrected spike-type (see Supporting Information, File S1 for more details). The current sample is divided hierarchically at the highest level into spring and winter growth habits and then within each growth habit by breeding population (Figure 1).

**Table 1:** Descriptive statistics by breeding populations

Breeding Program	Abbreviation	Spike-Type	No. Markers	Sample Size	Average Pairwise Diversity	Mean *F*_IS_	Private SNP
Spring barley			2392	2952	0.331		72
University of Idaho in Aberdeen	AB2	2	2103	239	0.266	0.983	3
	AB6	6	1791	142	0.257	0.984	1
Busch Agricultural Resources, Inc.	BA2	2	2029	172	0.203	0.997	0
	BA6	6	1770	147	0.166	0.997	0
Busch Agricultural Resources, Inc.(International)	BAI2	2	1508	60	0.295	0.997	0
University of Minnesota	MN6	6	1650	386	0.148	0.989	0
Montana State University	MT2	2	2041	317	0.264	0.989	1
North Dakota State University Two-row	N2	2	2177	353	0.241	0.959	5
North Dakota State University Six-row	N6	6	1722	380	0.197	0.98	2
Washington State University	WA2	2	2032	351	0.248	0.971	1
	WA6	6	1665	32	0.256	0.976	0
Winter barley			2336	661	0.291		16
Oregon State University	OR2	2	2052	73	0.293	0.960	0
	OR6	6	2272	268	0.274	0.968	11
Utah State University	UT2	2	1650	30	0.360	0.975	1
	UT6	6	2283	343	0.303	0.970	6
Virginia Polytechnic Institute and State University	VT6	6	2025	320	0.246	0.985	2
Average			1972.1	401.4	0.258	0.980	6.7

### Genotyping

All 3971 accessions were genotyped with 2882 Barley Oligo Pooled Assay Single Nucleotide Polymorphisms, referred to as BOPA SNPs (Close *et al.* 2009) using Illumina GoldenGate Technology (Illumina, San Diego, CA). BOPA SNPs were identified primarily from resequencing of expressed sequenced tags (ESTs) (Close *et al.* 2009). SNP order along each linkage group is based on the consensus genetic map of Muñoz-Amatriaín *et al.* (2011). Sampled accessions were self-fertilized to at least the F_4_ generation before genotyping (Hamblin *et al.* 2010; Wang *et al.* 2012), resulting in average expected heterozygosity of 6.25%. Genotyping information was downloaded from (http://triticeaetoolbox.org/) using only the OPA 2011 markers. The options to download the dataset were set to minimum MAF ≥0% and to remove markers and accessions with >99.9% missing data.

SNP annotations and metadata information, including gene names and whether SNPs occur in genic or nongenic regions, were obtained using SNPMeta (Kono *et al.* 2014).

### Quality control

The dataset was filtered for monomorphic SNPs, and SNPs or accessions with more than 25% missing data. Additionally, we removed accessions with incomplete sample information (*i.e.*, spike-type or growth habit) and accessions where single lines represented a population. We also removed accessions with heterozygosity that exceeded the expected 6.25%, a conservative threshold intended to filter out accessions that might have genotyping errors. Finally, we removed genetic stocks or near-isogenic lines such that one accession from each near-isogenic line set was retained in the dataset.

### Summary statistics

Descriptive statistics were calculated for each of the 16 breeding populations (Table 1). The degree of inbreeding was estimated by the inbreeding coefficient *F*_IS_ (1 – H_o_/H_e_) using a custom Python script. The similarity between samples was estimated by the percent pairwise diversity calculated using the *compute* program from the libsequence library (Thornton 2003). Monomorphic SNPs in each population were excluded and heterozygous and ambiguous calls were treated as missing data. The SharedPoly program from libsequence (Thornton 2003) was used to count the number of private SNPs in each breeding population.

### Derived site frequency spectrum (SFS)

Derived site frequency spectra (*i.e.*, the distribution of the derived allele frequencies) were calculated and plotted for each of the 16 populations and for the complete dataset. To infer ancestral state, SNP states from *Hordeum bulbosum* as reported for BOPA SNPs were used (Fang *et al.* 2014). This involved alignment of RNAseq data from one diploid accession of *H. bulbosum* (Cb2920/4) to the Morex draft assembly (Mayer *et al.* 2012), and calling the *H. bulbosum* nucleotide at the BOPA SNP positions. Ambiguous nucleotide calls, *trans*-specific polymorphisms, and sites at which *H. bulbosum* segregates for different nucleotides than barley (*H. vulgare* subsp. *vulgare)* were treated as missing data.

### Joint derived SFS

To compare the change in frequency of derived alleles between partitions of the data we used the joint derived SFS. Following the same procedure as for the derived SFS for each breeding population, we calculated the derived SFS within winter or spring accessions and within two- or six-rowed accessions using a custom R script. The derived SFS was compared between growth habit and between spike-types. The joint derived SFSs were plotted using the R package grDevices (R Core Team 2014).

### Population structure

The degree of differentiation among individuals from all breeding programs was estimated by Principal Component Analysis (PCA). The analysis was performed using the SmartPCA program from the EIGENSOFT package (Patterson *et al.* 2006). SmartPCA permits PCA analysis with SNPs that include missing data.

### Maximum likelihood tree of relatedness and migration

The population relatedness and patterns of gene flow between breeding populations was inferred using a maximum likelihood approach implemented in TreeMix (Pickrell and Pritchard 2012). To polarize the divergence among populations we used genotyping data for 803 landrace accessions from the Old World (Poets *et al.* 2015) (see https://github.com/AnaPoets/BarleyLandraces). The 2021 SNPs shared between datasets were used to build a population tree using landraces as the outgroup. We ran 25 replicates of the tree, bootstrapping with 75 SNP windows. We used the replicate with the lowest standard error for the residuals as the base tree topology and inferred the likelihood of having between one and five migration events among breeding populations. The plot of the residuals was used to evaluate which tree best fit the data. Candidates for admixture can be identified by those population pairs with residuals above zero standard error, which represent populations that are more closely related to each other in the data than in the best-fit tree (Pickrell and Pritchard 2012).

### Changes in allele frequency

To identify putative targets of long-term selection involved in the spike-type and growth habit differentiation among breeding programs we used the Weir and Cockerham (1984) measure of *F*_ST_ referred to as θ, as implemented in the R package hierfstat (Goudet 2005). *F*_ST_ was calculated for the following partitions of the data: (1) spring *vs.* winter, (2) two- *vs.* six-rowed, and (3) among breeding populations. Owing to the high level of inbreeding in the dataset, a haploid model for *F*_ST_ estimation was used as this better represented the sample size in our study. Heterozygous SNPs were treated as missing data. An empirical genome-wide threshold for the top 2.5% of *F*_ST_ values was used to identify SNPs with large differences in frequency relative to the genome-wide average. To identify the degree of differentiation that each breeding population has with respect to other populations we report *F*_ST_ for all pairwise comparisons. To determine the degree to which our *F*_ST_ results are robust to either the specific set of SNPs or individuals used here, we used bootstrapping to remove either 20% of SNPs or samples per iteration. We ran 100 iterations in each case.

To characterize average allele frequency divergence for breeding populations we used the *F*_ST_-like measure of Nicholson *et al.* (2002), reported here as “*c*.” This estimate differs from other *F*_ST_-like measures in that it attempts to estimate the degree of divergence of a population from ancestral allele frequencies. For this analysis, the dataset was divided into two groups: spring six-rowed and spring two-rowed. We do not report *c* values for winter barleys owing to limited sampling. For this analysis, monomorphic SNPs were removed from each group. The *c* value was calculated using the popdiv program from the popgen package in R (Marchini 2013). We used a burn-in period of 1000 iterations followed by a run length of 10,000 iterations with the scale parameter of a Dirichlet distribution used to update global allele frequencies *m* = 10 (see File S1 for more details).

### Analysis of resequencing data for known genes contributing to phenotypic differentiation

Resequencing data for 10 accessions for vernalization sensitivity loci (*Vrn-H3*, *Sgh3*) (von Zitzewitz *et al.* 2005) and 96 accessions for the *Vrs1* gene controlling spike-type differentiation (Komatsuda *et al.* 2007) were obtained from NCBI Popsets [UID #157652625 (Karsai *et al.* 2008) and 219664771 (unpublished data)]. Contextual sequences for SNPs known to occur in these genes were downloaded from T3. To determine the position of SNPs within these genes and their correlation with growth habit and spike-type differentiation, SNP contextual sequence for individual SNPs were aligned to each resequencing dataset in Geneious v.7.1.9 (Kearse *et al.* 2012).

### Identity by state

We used an IBS analysis to identify shared genomic segments among individuals between breeding populations potentially indicative of recent introgression. The analysis used PLINK v.1.90 (Chang *et al.* 2015) with window sizes of 50 and 100 SNPs, allowing for up to 10% mismatch. The frequency of shared segments between two populations was estimated for each SNP window. This analysis made use of phased genotyping data, with phase inferred using fastPHASE v1.2 (Scheet and Stephens 2006). Missing genotypic state was treated as missing (*i.e.*, genotypic state inferred during phasing was ignored). The phased data were only used for IBS and pairwise haplotype sharing analyses.

### Pairwise haplotype sharing

To explore recent events of selection within populations, we used the pairwise haplotype sharing (PHS) approach (Toomajian *et al.* 2006). A shared haplotype is defined as a genomic segment that extends out from a focal SNP, and is shared among individuals in a population. PHS is a form of IBS analysis that compares the extent of shared haplotypes among individuals normalized by genome-wide sharing. A PHS score depends on the length of the shared haplotype and its frequency in the population. Extended shared haplotypes are potentially suggestive of recent or ongoing selection, owing to limited potential for recombination to break down genomic regions subject to recent selection (Horton *et al.* 2012). PHS was calculated within each breeding population using a customized Perl script (Cavanagh *et al.* 2013). An empirical threshold of the upper 2.5% of PHS values and a minimum focal SNP frequency of 10% within each population were used to identify outliers in the distribution of PHS.

### Four-population test for gene flow detection

The robustness of patterns of migration inferred using TreeMix (see *Results* for details) was assessed using the four-population test (*f_4_*-test) (Keinan *et al.* 2007; Reich *et al.* 2009). The *f_4_*-test is designed to distinguish introgression from incomplete lineage sorting. The test evaluates trees of relatedness among populations and measures genetic drift along lineages quantitatively based on the variance in allele frequencies. Significant deviations from zero in three possible tree topologies (see File S1 for details) indicate that the tree evaluated does not fit the data, suggesting the presence of gene flow.

We used the topology inferred in TreeMix as our hypothesized relationship between populations. We inferred that a tree of relatedness with three migration events provides a better fit to the data than topologies that include either a lesser or greater number of migration events (see *Results* for more details). Following the [(A, B),(C,D)] notation for a tree topology with four populations (Figure S1), we assessed migration among the following sets of populations: [(N2, X),(Y_1_, Y_2_)], [(UT6, UT2), (Y_1_, Y_2_)] and [(OR2, X), (OR6,VT6)], where X was replaced iteratively for any two-rowed barley and Y_1_ and Y_2_ were replaced by a pair of six-rowed barley populations. The populations UT6, OR2, and N2 were chosen because of an indication of gene flow based on TreeMix results. Pairs of populations from the same branch of the TreeMix topology were considered on the inference that these populations are more similar to each other due to shared ancestral polymorphisms. The second pair of populations was selected from the branch containing the population putatively involved in the migration event (populations connected by the arrows in Figure 6). Since the population putatively involved in migration was not clearly defined, we ran the analysis iteratively between each possible pair of populations on the branch. We used the fourpop option in the TreeMix software (Pickrell and Pritchard 2012) to estimate the *f_4_-value* for each configuration. Significance of *f_4_-values* was determined at *P* < 0.05. We infer that migration has occurred when the three possible trees had a significant nonzero *f_4_-value*.

### Data availability

Table S1 contains information about the samples and Table S2 presents SNP ID numbers, genetic positions, and annotation information. The computer code used for all analyses (unless otherwise specified) and plots are available at https://github.com/MorrellLAB/NorthAmerica_Fst.

## Results

The original dataset included 3971 accessions genotyped with 2882 SNPs representative of 10 breeding programs across the United States. We removed 340 SNPs and 241 accessions with ≥25% missing data, 22 accessions with heterozygosity >6.25% across SNPs, 13 accessions with missing growth habit (spring *vs.* winter) or spike-type (two- *vs.* six-row spikes) information, 79 near-isogenic lines, and three accessions with single accessions representing a population. After quality control our dataset consisted of 3613 barley accessions (Table S1) and 2542 SNPs (Table S2).

### Characterization of North American breeding populations

Analysis of the structure of the North American populations using PCA (Figure S2) revealed that the primary population structure (PC1 = 19.6% variance) is explained by differences in spike-type, which corresponds to an average *F*_ST_ of 0.23 (Figure S3). PC2 indicates that 9.1% of the variance among lines is explained by differentiation in growth habit, with an average *F*_ST_ of 0.17. These results are congruent with earlier analyses on a subset of these populations (Cuesta-Marcos *et al.* 2010; Hamblin *et al.* 2010; Wang *et al.* 2012) and on a comparable set of samples analyzed by Zhou *et al.* (2012).

**Figure 2:**
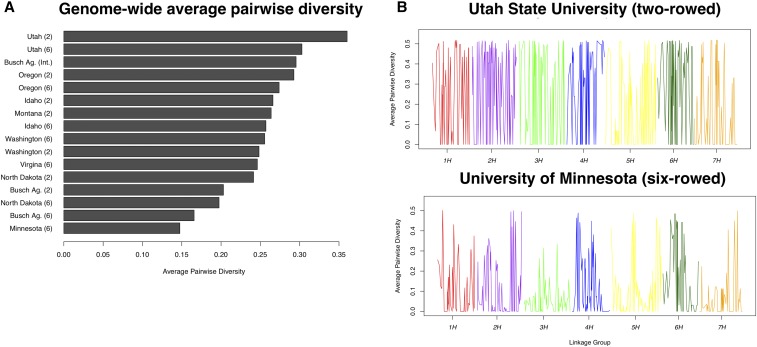
Genetic diversity in breeding populations. (A) Average pairwise diversity in each breeding population. (B) Genome-wide pairwise diversity for the most and least diverse populations according to average diversity, Utah State University (two-rowed) and University of Minnesota (six-rowed), respectively. Diversity values were averaged in 10-SNP sliding windows with a step of five SNPs.

The 16 breeding populations (*i.e.*, breeding programs separated by spike-type) were represented by an average sample size of 225 lines with a minimum of 30 lines (from UT2), and a maximum sample size of 386 for MN6 (Table 1 and Figure 1). On average, only two SNPs were private to each of the breeding populations, with a maximum of 11 private SNPs in OR6. Winter populations had 16 private SNPs with respect to spring populations, which in turn had 72 private SNPs (Table S3). The average inbreeding coefficient (*F*_IS_) across populations was 0.98 as expected after four generations of self-fertilization. Percent pairwise diversity ranged from 0.15 to 0.36 and averaged 0.25 across breeding populations (Table 1 and Figure 2A).

The joint unfolded SFS showed that there are slightly more rare variants segregating in spring than winter programs and slightly more rare variants segregating in six-rowed than in two-rowed programs (Figure S4), reflecting minimal impact of ascertainment bias in each of these partitions. In individual breeding populations there is an excess of rare and high-frequency variants relative to neutral expectations based on Kimura’s model of a population at equilibrium (Kimura 1983) (Figure S5). OR2, OR6, UT6, and UT2 have a higher proportion of midfrequency variants than other populations. When the breeding populations are considered jointly, the derived SFS (Figure S6) displays an elevated number of midfrequency variants, consistent with retention of variants segregating at an average minor allele frequency of 24% in the discovery panel (Close *et al.* 2009).

### Genome-wide scan for evidence of selection in North American breeding programs

**Figure 3:**
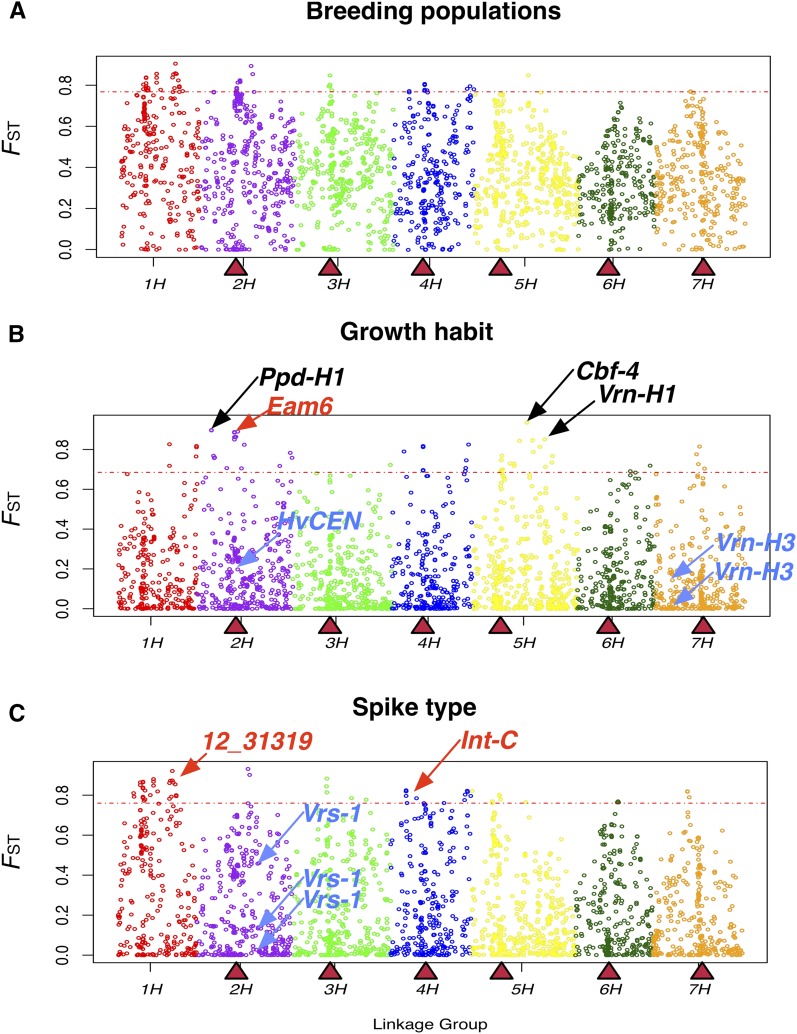
Allele frequency differentiation between comparisons of partitions of the data. (A) Among breeding populations; (B) between spring and winter types; and (C) between six- and two-rowed spike-types. Triangles indicate the genetic position of centromeres (Muñoz-Amatriaín *et al.* 2011). Dotted line corresponds to the 97.5th percentile of the *F*_ST_ values distribution. Linkage groups are shown with different colors. In black font are genes corresponding to outlier *F*_ST_ SNPs. In red font are either the SNP linked to a predicted gene involved in the phenotype or the gene itself. The arrows below the threshold line indicate SNPs located in genes known to be involved in the phenotype.

The distribution of *F*_ST_ statistics for comparisons of growth habit, spike-type, and breeding populations showed that among the three classifications, the greatest differentiation in allele frequencies is found among breeding populations with an average *F*_ST_ of 0.37, *vs.* 0.23 for spike-type and 0.17 for growth habit (Figure 3 and Figure S3). For each of the three partitions, we identified 61 SNPs in the upper ≥0.975 of *F*_ST_ values (Table S4, Table S5, and Table S6). Of these, 34 SNPs were outliers for both the spike-type and breeding population comparisons, seven were common between breeding populations and growth habit comparisons, and there were no common outlier SNPs between growth habit and spike-type comparisons.

Bootstrapping, by removing 20% of individuals in each partition of our sample or 20% of SNPs, was used to determine whether *F*_ST_ outlier detection was dependent on the particular individual’s sampled or set of markers used. Across samples we recovered an average of 95% of SNPs outliers in any given comparison. Outliers include SNPs near or in genes mentioned below. Across SNPs we recovered an average of 87% of the genetic regions where SNPs were identified as outliers in the full dataset. This average excludes linkage group 1H where the large number of outliers precludes identification of outliers plausibly attributable to selection

### Known genes contributing to phenotypic differentiation

We identified a SNP outlier (12_30883, linkage group 5H) in the *F*_ST_ comparison for growth habit located in the vernalization sensitivity locus (*Vrn-H1*, *Sgh2*) (von Zitzewitz *et al.* 2005) known to be involved in the growth habit differentiation. SNPs within two additional well-characterized genes including photoperiod response-1H (*Ppd-H1*, *Eam1*) (Turner *et al.* 2005; Jones *et al.* 2008) and c-repeat binding factor 4 (*Cbf4*) (Haake *et al.* 2002) were also found to be *F*_ST_ outliers (Figure 3B). Both of these genes have reported functions related to growth habit differentiation. *Ppd-H1* alters the flowering time response under long-day conditions, thus making it possible to avoid unfavorable seasonal conditions (Lister *et al.* 2009) whereas *Cbf4* contributes to cold acclimation (Skinner *et al.* 2005).

While vernalization sensitivity and photoperiod response are important determinants that contribute to growth habit through an environmental response, there are also loci that contribute to flowering time independent of environmental cues. This class of genes in barley is referred to as Earliness *per se* (EPS) loci; one characterized example is the early maturity 6 locus (*Eam6*, *eps2S*, *mat-c*) (Boyd *et al.* 2003; Comadran *et al.* 2012). A linkage mapping study placed early maturity 6 locus (*Eam6*) (Laurie *et al.* 1995) near the centromere of linkage group 2H (cM 67.8), a region where we identify six SNPs with elevated *F*_ST_ (average *F*_ST_= 0.87) (see Table S5 and Figure 3). The two SNPs (11_20438 and 11_20366) most strongly associated with flowering time in a recent GWAS study (Comadran *et al.* 2011) that resulted in the cloning of the barley *Centroradialis* gene (HvCEN) responsible for flowering time variation (Comadran *et al.* 2012) were not identified as outliers in our *F*_ST_ comparison of growth habit. However, both SNPs 11_20438 and 11_20366 have an above average *F*_ST_ of 0.52 and 0.25, respectively.

In an association mapping (AM) study Cuesta-Marcos *et al.* (2010) identified two significant SNPs in linkage groups 1H and 2H (12_31319 and 11_10213, respectively) associated with spike-type differentiation. In our analysis, 12_31319 has an outlier *F*_ST_ value of 0.86, while 11_10213 has an above average *F*_ST_ = 0.47 but is not defined as an outlier (Figure 3C). Additionally, there are two SNPs (11_20422 and 11_20606) in linkage group 4H that have been identified near the *Intermedium-C* gene (*Int-c*) (Ramsay *et al.* 2011), which is a modifier of lateral spikelet development in barley. After quality control, only 11_20422 remained in our dataset. This SNP has an *F*_ST_ value of 0.81 and is thus considered an outlier in the comparisons between spike-types (Figure 3C).

Other SNPs occurring in well-characterized genes did not appear as outliers despite the previously reported contribution to function. There were three SNPs in our dataset (12_30893, 12_30894, and 12_30895 on linkage group 7H) occurring within the vernalization sensitive locus 3 (*Vrn-H3)* (Yan *et al.* 2006) that were not outliers in the *F*_ST_ comparison between spring and winter types (Figure 3). This gene is an ortholog of the *Arabidopsis thaliana* flowering locus T (*FT*) that promotes flowering time under long days (Turck *et al.* 2008). In barley, nine linked polymorphisms in the first intron have been predicted to be responsible for the variation in flowering time at this locus (Yan *et al.* 2006). Our alignment of the three outlier SNPs to resequencing data of this gene from 10 barley accessions (Karsai *et al.* 2008) identified three of the SNPs segregating in the first intron of *Vrn-H3* (Figure S7A). In a study by Yan *et al.* (2006) nucleotide states “A” and “G” in 12_30894 and 12_30895, respectively, were associated with spring barley types (two accessions), while “T” and “C” were associated with winter types (eight accessions). The resequencing data in part support this association having two out of four spring barleys with the inferred haplotype while five out of six winter barleys carried the correct inferred haplotype. However, in our much larger dataset of spring and winter accessions these SNPs are segregating at an average allele frequency of 50% in both spring and winter growth habits (Figure S7B), showing no association between these SNPs and spring *vs.* winter growth habit (maximum *F*_ST_ = 0.04).

The *Vrs1* gene, a well-characterized contributor to spike-type (Komatsuda *et al.* 2007) was not identified in the *F*_ST_ comparison between two- and six-rowed accessions. Our SNP panel included five SNPs (12_30896, 12_30897, 12_30899, 12_30900, and 12_30901, linkage group 2H) in *Vrs1*. However, none of the SNPs reached the empirical cutoff for *F*_ST_ in this comparison, with a maximum observed *F*_ST_ of 0.45. The “G” nucleotide state at SNP 12_30900 results in an amino acid substitution associated with the six-rowed phenotype (*vrs1.a3* allele); however, the alternative nucleotide state “C” can be found in either six- or two-rowed accessions (Komatsuda *et al.* 2007; Youssef *et al.* 2012). In a panel of 96 European accessions of cultivated barley (Popset ID 219664771) (Figure S7B) the “G” state always resulted in a six-rowed phenotype. In our sample of North American breeding programs 2% of individuals that carry this variant state were reported as two-rowed barleys, which is similar to previous results that found this SNP significant for spike-type differentiation segregating in two-rowed accessions with an allele frequency of 1% (Cuesta-Marcos *et al.* 2010). The “C” state segregated at 50% frequency in each of the row-type partitions.

### Haplotype sharing and evidence for recent selection

The PHS analysis permits the identification of genomic regions that are putatively involved in more recent selection. PHS analysis within individual breeding populations identified a total of 775 SNPs in the upper ≥0.975 of the PHS distribution (Table S7 and Figure S8). In a small number of cases, focal SNPs in the PHS analysis were identified as outliers in more than one breeding population. Sharing of PHS outliers is greatest for OR6 and UT6 (22), BA6 and WA6 (17), and AB2 and OR2 (14) (Table S8). The number of focal SNPs that were PHS outliers shared among populations were an average of three SNPs shared within two-rowed populations and four SNPs within six-rowed populations. An average of two focal SNPs were shared between two- and six-rowed populations. Out of the 775 SNPs, 77 were in genes with known function. The haplotypes for these significant PHS values varied in length from 19.7 cM to 139.6 cM with mean 55.9 cM across breeding populations (Figure S9A and Table S9). The frequency of the SNP state with significant PHS ranged from 10% (the minimum value we consider) to 61% with mean 24% (Figure S9B).

Within BAI2, BA6, MN6, UT2, UT6, WA6, and OR2 we observed long runs of haplotype sharing (average length 112.45 cM) at an average frequency of 24%, significantly exceeding genome-wide similarities. These regions were putatively subject to recent selection. Among outliers for PHS, BA2 showed significant PHS surrounding three SNPs (12_30893, 12_30894, 12_30895, in linkage group 7H) with all three SNPs occurring within *Vrn-H3*. AB2, MT2, OR2, and OR6 had an outlier PHS value for SNP 12_30901 (linkage group 2H) in the *Vrs1* gene. The SNPs in *Vrn-H3* and *Vrs1* were at an average frequency of 22.25% in each of these populations, with an average length of shared haplotype of 66.98 cM (Table S7 and Table S9). There are three SNPs (12_20368, 12_20593, and 12_21049 in linkage group 2H) with significant PHS value in OR2 (frequency 11% and haplotype length 138.9 cM). SNPMeta annotations identified SNP 12_20593 within the nicotianamine synthase 2 (*nashor2*) gene in barley, which is a key enzyme for iron homeostasis (Herbik *et al.* 1999), and SNP 12_20368 within a gene with sequence similarity to galactinol synthase 2 gene in wheat (*TaGolS* 2), which in turn is orthologous to the characterized gene *TaGolS* 2 in *A. thaliana* (Taji *et al.* 2002). In *A. thaliana*, *TaGolS* 2 has been identified as playing an important role in drought-stress tolerance (Taji *et al.* 2002).

### Divergence from ancestral allele frequencies

**Figure 4:**
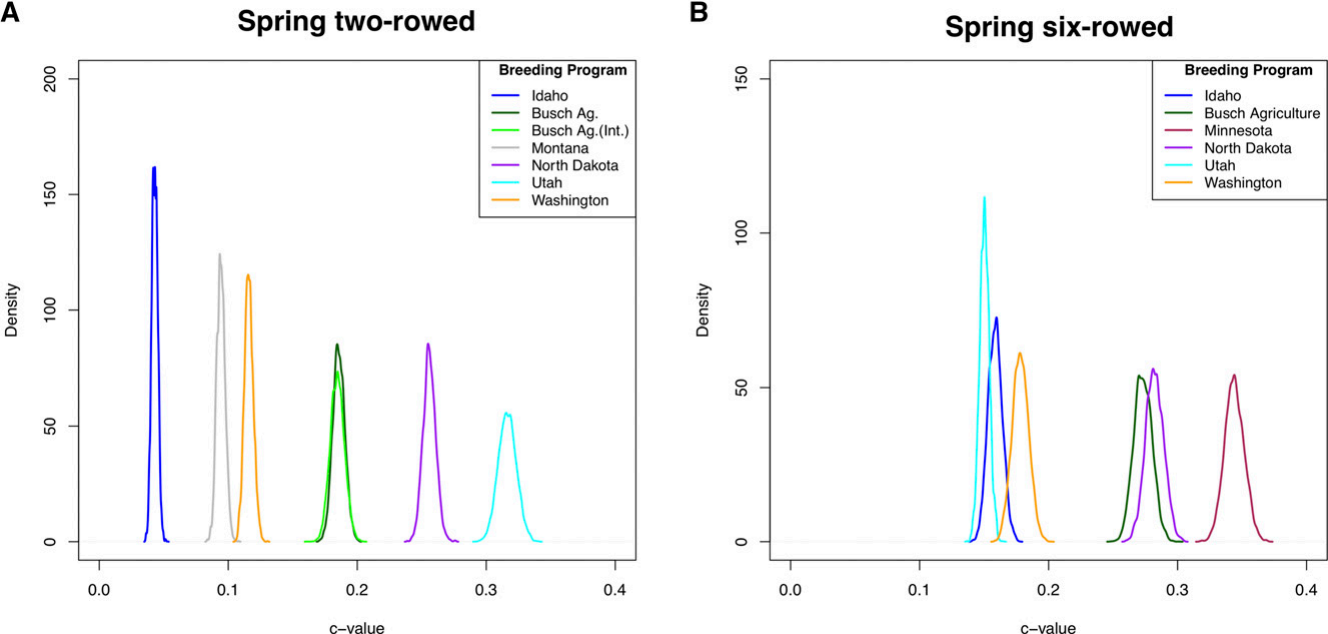
Marginal posterior density plots obtained for the *c* estimator for spring barley populations. Density distribution after 10,000 iterations. Plot shows the amount of drift estimated from ancestral allele frequencies (0,0 coordinates). (A) Two-rowed barley populations; (B) six-rowed barley populations.

Quantification of allele frequency divergence based on *c* (Nicholson *et al.* 2002) showed that six-rowed breeding populations have experienced more divergence than two-rowed populations, with mean *c* of 23.1% and 17.1%, respectively (Figure 4 and Table S10). Among the two-rowed populations, Utah two-rowed has diverged the most while Idaho two-rowed resembles ancestral allele frequencies, suggesting reduced effects of drift or linked selection in the latter population. Among the six-rowed populations, Utah six-rowed was the closest to ancestral allele frequencies while Minnesota six-rowed has experienced the most divergence. Utah two-rowed diverged more from ancestral allele frequencies than its six-rowed counterpart (contrasting the pattern observed in other spike-type pairs of populations). This is expected because the two-rowed individuals came from crosses between two-rowed and six-rowed varieties (data not shown).

### Gene flow between breeding populations

**Figure 5:**
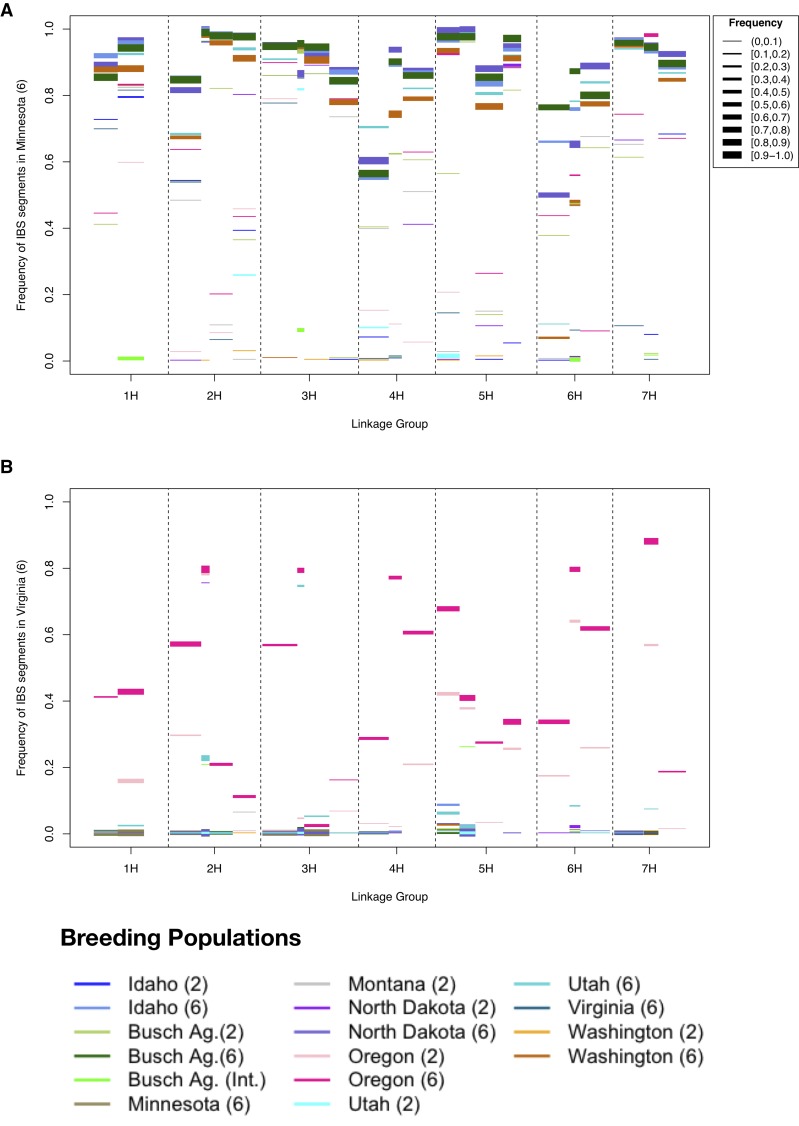
Frequency of IBS haplotypes in breeding populations from (A) University of Minnesota and (B) Virginia Polytechnic Institute and State University. Using 100 SNP windows. Linkage groups are in the X-axis. The Y-axis is the frequency of the haplotype in the population indicated in the Y-axis label (“base population”). The color of the bars in the plot represents the distinct barley breeding populations that share the same segment with the “base population”. The width of each bar corresponds to the frequency of that haplotype in the compared populations. The depiction shows that there is a high frequency of shared haplotypes among six-rowed spring barleys. This contrasts with the Virginia population (winter six-rowed) which shares a few chromosome segments only with Oregon six-rowed and to a lesser extent Oregon two-rowed.

To identify genomic segments subject to recent introgression, we tested for regions with a high degree of IBS between breeding populations. Window sizes of 50 (0.63–44.96 cM) and 100 (13.0–62.25 cM) SNPs were used, and we permitted up to 10% mismatch among haplotypes. As expected, populations within growth habit and spike-type had a higher degree of haplotype sharing than between these partitions. The degree of haplotype sharing was lower within spike-type than within growth habit (Table S11, Table S12, Table S13, Figure S10, and Figure S11). There was a high frequency of shared haplotypes among two-row populations at 50 SNP windows, but this was reduced when 100 SNP windows were considered. At 100 SNP windows BAI2 and N2 presented the lowest degree of shared haplotypes with other two-rowed populations, while BA2 shared the most haplotypes (Figure S11, C, E, and H). For both 50 and 100 SNP windows, high-frequency shared haplotypes were common for all six-rowed populations – see for example Figure 5A, except for the Utah populations (Figure S10, Figure S11, Table S14, and Table S15). This contrasted with VT6, which showed a very low degree of haplotype sharing, particularly with the various spring breeding populations (Figure 5B). Haplotype sharing for VT6 occurred primarily with the OR2 and OR6 populations, *i.e.*, with the only other populations with similar growth habit. However, OR2 and OR6 showed higher levels of allele frequency similarity with other breeding populations than VT6 (Figure S10, J, K, and N). These results are consistent with differentiation in allele frequency observed in *F*_ST_ comparisons between breeding populations (Table S16).

For 50 SNP windows, there were a high number of IBS segments shared between spike-types. However, when windows of 100 SNPs were considered, the majority of the IBS haplotypes were at low frequency (<20%) in one of the two populations compared (Table S13 and Table S14). An exception to this was the comparison between OR2 and OR6, where shared haplotypes were quite common. Sharing occurred at every 100 SNP window, with shared haplotypes at frequencies as high as 90% in the two populations (Figure S11K) with an average frequency within populations of 0.46 and 0.58 (for OR2 and OR6, respectively) (Table S15). IBS with 50 SNP windows for N2 and N6 spanned 94% of all windows, with shared haplotypes occurring at average frequencies of 22% and 50%, respectively (Figure S10 and Table S14). Increasing the window size to 100 SNPs identified many fewer shared haplotypes between N2 and N6, leaving 70% of the genome shared at an average frequency of 8% in N2 and 35% in N6 (Figure S11 and Table S15).

### Maximum likelihood tree of relatedness and migration

**Figure 6:**
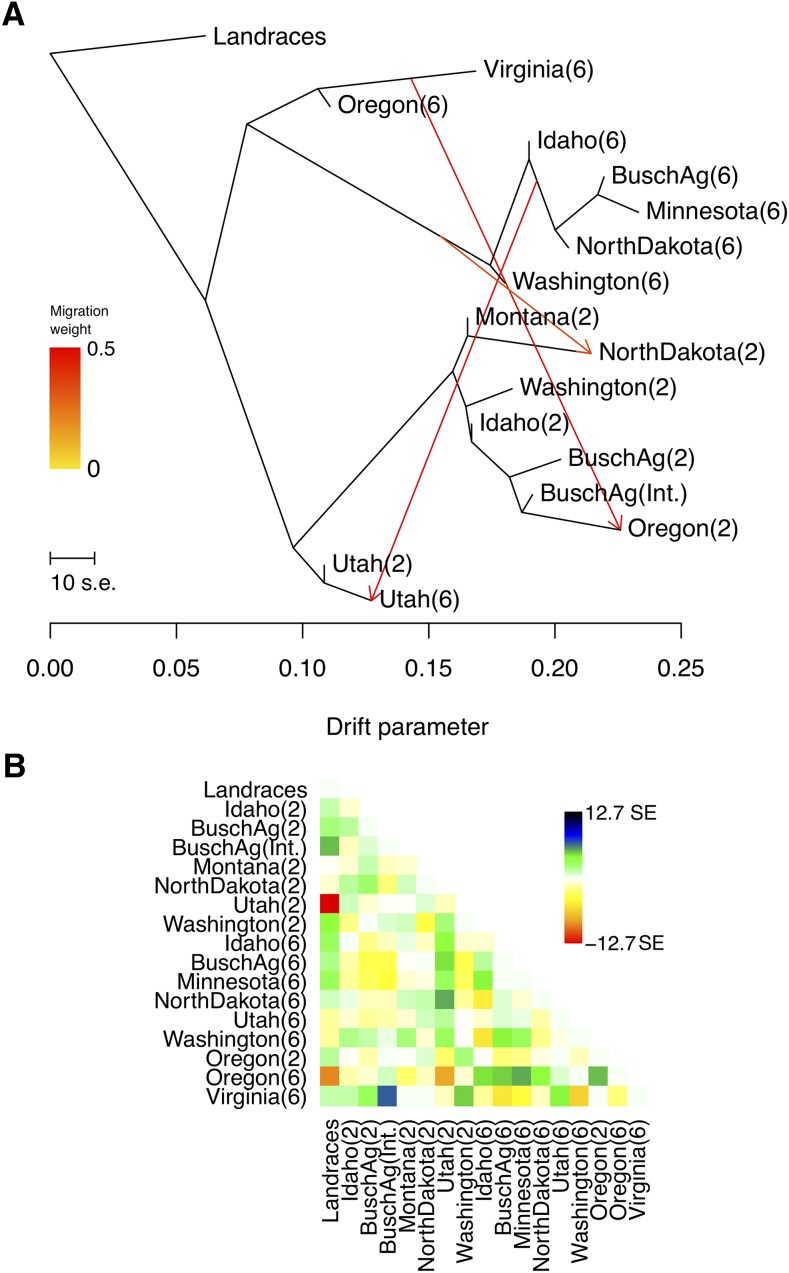
Tree of relatedness among North American breeding populations. (A) Plotted is the structure of the graph inferred by TreeMix for the 16 North American breeding populations, allowing three migration events. The arrows (migration events) are colored according to their weight. Horizontal branch length is proportional to the amount of genetic drift in the branch. The scale bar on the left shows 10 times the average standard error of the entries in the sample covariance matrix. (B) Residual fit of inferred tree of relatedness. Residuals above zero represent populations that are more closely related to each other in the data than in the best-fit tree, thus are considered candidates for admixture events. Spike-type for each population is shown in parentheses.

We determined that the tree topology that best describes the 16 populations separates the populations first by spike-type followed by growth habit (Figure 6), consistent with the *F*_ST_ and PCA results, with the exception of UT2 and UT6 that are not separated by spike-type. In the TreeMix analysis, the two Utah and winter populations were more similar to ancestral allele frequencies, while MN6 was the most diverged.

Adding three migration events resulted in the lowest residuals and standard errors relative to trees with no migration, or one, two, four, or five migrations (Figure S12). With three migrations, we infer exchange between spring six-rowed populations and N2; VT6/OR6, and OR2, and spring six-rowed populations and UT6 (Figure 6).

Based on the *f_4_*-test, six different topologies yielded significant results suggesting gene flow between UT6 and spring six-rowed populations (Table S17), with z-scores significantly different from zero (significance at *P* < 0.05). The *f_4_*-test for introgression between N2 and any spring six-rowed population resulted in values significantly different from zero, consistent with gene flow. The *f_4_*-test did not support introgression between OR2 and VT6 and/or OR6 since none of the possible tree topologies involving these populations were significant.

## Discussion

Comparative analyses of allele frequency differentiation and extended haplotype sharing in a sample of 3613 barley accessions representing 16 barley breeding populations result in five primary conclusions: (i) barley breeding programs in North America have strongly differentiated allele frequencies among breeding populations; across programs, inflorescence type followed by growth habit account for the greatest proportion of variance in allele frequency; (ii) a number of loci previously identified as major contributors to growth habit adaptation in barley (primarily in Europe) are readily identifiable as outliers in allele frequency in our *F*_ST_ analyses of North American breeding populations; (iii) we identify putative signals of recent and long-term selection similar in magnitude to those at previously isolated genes of known function; (iv) the average per SNP allele frequency divergence at the population level appears consistent with the dominant action of genetic drift and linked selection; and (v) we identify populations that are recently subject to exchange of genetic material. There are low levels of genetic exchange across spike-type boundaries.

### Identification of loci putatively subject to recent and long-term selection

Allele frequency differentiation sufficient to be detected in an *F*_ST_ outlier analysis is generally the result of long-term directional selection (Beaumont and Balding 2004). The SNPs identified as outliers for spring *vs.* winter growth habit include previously isolated genes of large effect, for example *Vrn-H1* and *Ppd-H1*, congruent with a previous *F*_ST_ comparison in European barleys (Comadran *et al.* 2012). Allele frequency differentiation between spike-type, which explains a larger portion of the allele frequency divergence in our sample, identified two SNPs in the genomic region of genes contributing to spike-type; however, SNPs within the well-characterized *Vrs1* locus were not identified as *F*_ST_ outliers (see below in the *Limitations of allele frequency comparisons* section). Many additional *F*_ST_ outliers are newly identified as putative targets of selection.

Despite a relatively recent introduction to North America, multiple genes found to contribute to agronomic traits in Eurasia appear to recapitulate allele frequencies in current, highly structured breeding programs. This appears to be particularly true when the phenotype is conferred by a single allele as opposed to an allelic series, as occurs at *Vrs1* where the six-rowed phenotype is conferred by multiple alleles (see Komatsuda *et al.* 2007). Perhaps more surprising is that many of the same allele frequency outliers are identified in European and North American samples despite very different demographic and breeding histories.

PHS analysis in the North American breeding programs detected a series of loci that are putatively targets of recent selection within barley breeding populations, comparable to findings in wheat (Cavanagh *et al.* 2013). The length of the haplotypes shared (average 81.38 cM) and their frequencies (average 25%) are consistent within recent generations, limiting the potential for recombination to break down the haplotypes. This analysis indicates that selection and linked selection are altering patterns of haplotype diversity across the genome. However, there are some chromosomes that have little or no evidence of selection in recent generations (*e.g.*, linkage group 2H in BA2 and WA2; see Figure S8).

### Limitations of allele frequency comparisons

Despite having five SNPs positioned in the *Vrs1* gene (controlling the fertility of lateral spikelet) (Komatsuda and Mano 2002), including one SNP (12_30900) variant cosegregating with the six-rowed phenotype, the comparison of allele frequencies differentiation between spike-type fails to identify outliers in *Vrs1*. One important contributing factor may be that the six-rowed phenotype can result from multiple disruptions of the *Vrs1* gene (Komatsuda *et al.* 2007). The phenomenon of multiple mutational paths to the same phenotype has been identified as “functionally equivalent mutations” (Ralph and Coop 2010) and can result in multiple variants at modest frequency controlling a phenotype. The maintenance of alleles of large effect at modest frequency in the population reduces the power to detect phenotypic associations, in part because any given variant explains a small portion of phenotypic variation (Thornton *et al.* 2013).

The BOPA SNP platform used here is largely derived from variants identified from cDNA libraries, from cultivated accessions (Close *et al.* 2009), and thus is highly enriched for SNPs in genic rich regions (Kono *et al.* 2014). The BOPA SNPs are 89% synonymous or noncoding and 11% are nonsynonymous (data not shown). However, SNPs genotyped within a locus can have limited correlation with a phenotype, depending on the haplotype on which they occur (see Nordborg and Tavaré (2002) for discussion of this issue). This may be the case for the previously cloned *Vrn-H3* where we see limited correlation between SNPs in this gene and allele frequency difference in the growth habit comparison. It should also be noted that the resequencing panel in which *Vrn-H3* alleles were found to be associated with the phenotype included only five spring and eight winter individuals (Yan *et al.* 2006). It is perhaps not surprising that sampling error (*i.e.*, small sample sizes) accounts for inconsistencies in the phenotype–genotype associations reported in previous studies and our study using a panel of >3000 accessions.

### The effects of linked selection and drift in breeding populations

The derived SFS in individual breeding populations identifies an excess of rare and high-frequency derived variants (Figure S5) with respect to neutral model of a population at equilibrium. During selection, alleles linked to the target variant can be carried to high frequencies. Therefore, an excess of rare and high-frequency derived variants can be associated with the effects of linked selection (Fay and Wu 2000) as has been observed in a resequencing study of *Oryza sativa* (Caicedo *et al.* 2007). The PHS results presented here suggest considerable potential to detect the effect of linked selection, particularly when the selection was recent, impacting large genomic regions. One of the largest genomic regions detected as an outlier in the PHS analysis is found in OR2; this haplotype involves 138.9 cM on linkage group 2H, observed at 11% frequency. Individuals carrying this haplotype were derived from a two- by six-rowed cross (Merlin × Strider) and were selected to recover the two-rowed phenotype (P. Hayes, personal communication). This resulted in a long shared haplotype centered on the *Vrs1* gene contributing to spike-type (identified by SNP 12_30901). Two of the SNPs that permitted the identification of the outlier haplotype occur within an iron homeostasis (*nashor 2*) and drought-stress tolerance genes (*TaGolS 2*).

Our comparisons of allele frequency differences among individual barley breeding populations (see Figure 4 and Table S16) suggest that genetic drift (and likely linked selection) plays a major role in differentiation among these populations. One major factor that can reduce effective population size, and thus increase the role of drift in breeding populations, is the frequent reuse of popular cultivars as parents in breeding programs. Historically, this included founders such as Betzes, Hanna, Lion, Manchuria, and Trebi (Martin *et al.* 1991) but more recently lines such as Bonanza, Cheri, and Chevron occur frequently in pedigrees (Condón *et al.* 2008).

The cumulative effects of selection and genetic drift over several generations of breeding may result in reduced response to selection (Hanrahan *et al.* 1973), with larger effects of drift when the population is small and/or highly inbred (Robertson 1960). The magnitude of drift suggests that the majority of barley breeding programs have small effective population sizes. Gerke *et al.* (2015) noted that genetic drift played a major role in changes in allele frequency over the history of maize breeding in North America. Although, in the larger partitions of barley populations, signals of directional selection are clearly evident at loci of large effect, drift may be extremely important to the loss of variation for variants of small effect (or for phenotypes determined by multiple genes), especially when the selective pressure is sporadic (*e.g.*, drought tolerance).

### Gene flow among North American breeding populations

Gene flow between populations can be detected by the presence of large chromosomal regions in (admixture) linkage disequilibrium (Chakraborty and Smouse 1988; Chakraborty and Weiss 1988; Briscoe *et al.* 1994) or extended genomic regions of shared ancestry (Gusev *et al.* 2009, 2012). Shorter shared haplotypes indicate shared ancestry a larger number of generations before present (more generations of recombination). Considering the high frequency of IBS segments (and associated low *F*_ST_) across the genome between populations of similar inflorescence type, we speculate that these segments could reflect a history of shared ancestry among these populations, possibly predating the separation of breeding programs (Martin *et al.* 1991).

Gene flow that involves adaptive variation can result in differential retention of genomic segments in the recipient population, and can also be evident as shared genomic regions with reduced diversity (Hufford *et al.* 2013). For example, MN6 demonstrates a high degree of IBS with other six-rowed populations (Figure 6A), and these genomic regions have lower average pairwise diversity than other regions when the six-rowed populations are analyzed together (Figure S13).

With regard to IBS, VT6 is the most clearly differentiated from other North American populations, showing similarity only to the other winter populations, OR2 and OR6 (for 50 SNP windows, Figure S10). Pairwise *F*_ST_ with other breeding populations and genetic differentiation as measured by PCA (Figure S2B) suggest that this isolation has been maintained over many generations. Large IBS segments (100 SNP windows) shared between VT6 and OR2 and OR6 (Figure 5B) indicate that winter populations have recently exchanged genetic material. The demographic history inferred in the TreeMix analysis supports this relationship (Figure 6), particularly when invoking three migration events, the topology best supported by the data. However, the formal examination of migration using the *f_4_*-test failed to support gene flow among two- and six-rowed winter populations, thus suggesting that haplotype similarities across spike-types are due to ancestral history rather than recent introgression. In addition to the postulated shared ancestry, genetic similarity is expected between these populations under the premise that both Virginia and Oregon breeding populations have been subject to relative recent introgression from leaf rust resistance lines from the International Maize and Wheat Improvement Center (CIMMYT) (C. Griffey and P. Hayes, personal communication). Isolated populations, like VT6, can carry adaptive variants absent in other populations (Kristiansson *et al.* 2008). Therefore, VT6 could be used for the identification of new variants for yield or disease resistance, as well as a source of genetic variation to increase diversity in other breeding populations.

The large number of IBS segments shared between the Utah populations and most of the six-rowed populations yielded a significant signal of introgression based on the *f_4_*-test. It is possible that the signal detected here is due to the ongoing genetic exchange between UT2 and UT6 (as documented in pedigrees) since 2001 (D. Hole, personal communication) and the genetic history shared between six-rowed barleys. This may also account for the elevated pairwise diversity in the Utah populations. Additionally, there is evidence of introgression between N2 and two six-rowed populations (WA6 and AB6), but not with N6. Introgression between the six-rowed populations and N2 are uncommon (J. Franckowiak, personal communication), thus this result needs more exploration.

Using the tree of relatedness as our hypothetical relationship, a necessary component of the *f_4_*-test, forces populations of the same spike-type to be assumed as related by shared history. Therefore, gene flow within spike-types is not investigated. Although crosses across spike-type are not common in barley breeding programs due to concerns of recombining desirable alleles with less favorable ones, introgression across spike-type could increase diversity (Martin *et al.* 1991). We detect two instances of introgression across spike-type that result in higher levels of genetic diversity.

### Caveats of the analysis

It is important to note that the results presented here are dependent on the samples submitted for genotyping (https://triticeaetoolbox.org) by each breeding program. Given that one of the goals of the Barley Coordinated Agricultural Project (http://www.barleycap.org) was to evaluate the diversity in the North American barley breeding program, each program was encouraged to submit representative lines. We assume that the data represent the diversity within each breeding program and that the sampling scheme across breeding programs is comparable. Deviations from these assumptions could influence the results in four primary ways: (i) underestimation of the diversity within breeding populations; (ii) overestimation of the role of drift in breeding populations due to reduced representation of the parental lines used in the breeding programs; (iii) over- or underestimation of allele frequency differentiation between partitions of the data; and (iv) excess of shared haplotypes due to accessions highly related by pedigree (*e.g.*, sibs and half-sibs). With these caveats in mind, we made the best use of the dataset to uncover the underlying genetic response to breeder’s efforts. We encourage readers of this paper and barley breeders to evaluate the results with an understanding of the limitations of the sampling.

### Implications

Comparative population genetic approaches have the potential to uncover breeding history at a level of detail not previously possible. Our applications of allele frequency differentiation analyses in barley breeding populations are able to identify candidate genes or linked markers controlling major traits. Though the nature of the target of selection identified by individual SNPs is not always readily apparent, the identification of *F*_ST_ outliers reported here provides a reasonable first step toward the discovery of genes underlying agronomic adaptation or potential markers linked to these genes.

*F*_ST_ analyses also reveal the effects of linked selection and drift on breeding populations. Linkage has the potential to reduce the efficacy of selection by forcing selection to act on the net effect of linked variants (Hill and Robertson 1966; Robertson 1970). Furthermore, beneficial variants could be in linkage with deleterious mutations (Felsenstein 1974). Gains from selection in breeding programs will depend on the disassociation of unfavorable linkage (Morrell *et al.* 2012; Rodgers-Melnick *et al.* 2015). This can be achieved by increasing the effective amount of recombination. Hill and Robertson (1966) suggested that a generation of relaxed selection with a larger number of contributing parents between each generation of selection could increase the amount of recombination in the population, increasing the potential to disassociate these variants.

## Supplementary Material

Supporting Information
