# Supplementary material for: The Effects of Both Recent and Long-Term Selection and Genetic Drift Are Readily Evident in North American Barley Breeding Populations
Source: G3 (Bethesda). 2015 Dec 29;6(3):609–22. doi: 10.1534/g3.115.024349 (PMC4777124; doi:10.1534/g3.115.024349)
Supplement: Supporting Information [file supp_6_3_609__index.html]

The Effects of Both Recent and Long-Term Selection and Genetic Drift Are Readily Evident in North American Barley Breeding Populations — Supporting Information 

# The Effects of Both Recent and Long-Term Selection and Genetic Drift Are Readily Evident in North American Barley Breeding Populations

## Supporting Information for Poets *et al.*, 2016

**Files in this Data Supplement:**

- File S1 - Plant materials (.pdf, 116 KB)
- Figure S1
- Figure S2
- Figure S3
- Figure S4
- Figure S5
- Figure S6
- Figure S7
- Figure S8
- Figure S9
- Figure S10
- Figure S11
- Figure S12
- Figure S13
- Table S1
- Table S2
- Table S3
- Table S4
- Table S5
- Table S6
- Table S7
- Table S8
- Table S9
- Table S10
- Table S11
- Table S12
- Table S13
- Table S14
- Table S15
- Table S16
- Table S17
