# Supplementary material for: The Effects of Both Recent and Long-Term Selection and Genetic Drift Are Readily Evident in North American Barley Breeding Populations
Source: G3 (Bethesda). 2015 Dec 29;6(3):609–22. doi: 10.1534/g3.115.024349 (PMC4777124; doi:10.1534/g3.115.024349)
Supplement: Supporting Information [file supp_g3.115.024349_FileS1.pdf]

FILE S1

Plant materials

We noted that 10 samples from Oregon (STAB7-079, OR76-071, F6-1, F6-2, F6-3,F6-4,F6-6,F6-7,F6-8 and G388) were mislabeled in the T3 data base as two-rowed (P Hayes, personal communication). In this manuscript we classified them correctly as six-rowed type. There were three accessions removed from the data set because they where the only ones representing a population: one accession from Montana and two accessions from Oregon two-rowed.

Nicholson's "*c*"

This approach uses a Markov Chain Monte Carlo model (MCMC) to estimate allele frequency changes at each population from a comparison to a common ancestral allele frequency (Nicholson *et al.* 2002). The model explicitly includes the SNP ascertainment process and corrects allele frequency differentiation influenced by it. The differences in allele frequencies using *c* are assumed to be the results of demographic events rather than selection, however the fitting of the model could result in the discovery of loci targets of selection (Nicholson *et al.* 2002). The model assumes a star phylogeny in which populations split and evolve in subsequent isolation under drift alone.

Four population test

The idea of the f_4*-test* is that for each set of four populations A, B, C, and D there are three possible unrooted trees that best describe the relationship in the absence of gene flow: ((A,B),(C,D)), ((A,C),(B,D)), and ((A,D),(B,C)). If the assumed topology is ((A,B),(C,D)) the f_4 statistic is calculated as the product of the difference of allele

frequencies between A and B, and between C and D. Thus for one SNP $f_4 = (p_A - p_B) \times (p_C - p_D)$. Genome-wide the f_4 is just the mean of the *f_4-values* at every SNP. If the tree topology was correct, then the allele frequency between pairs of populations should be uncorrelated resulting in $f_4 = 0$. Significant deviations from zero indicate that the tree evaluated does not fit the data. The significance of deviations from zero was assessed by calculating a normally distributed Z-score, using a Block Jackknife to obtain a standard error correcting for linkage disequilibrium among SNPs. Having three trees being significantly different from zero implies that the relationship between the populations compared is more complex, suggesting the presence of gene flow between the populations involved in the trees.
