# Supplementary figures and images for: The Effects of Both Recent and Long-Term Selection and Genetic Drift Are Readily Evident in North American Barley Breeding Populations
Source: G3 (Bethesda). 2015 Dec 29;6(3):609–22. doi: 10.1534/g3.115.024349 (PMC4777124; doi:10.1534/g3.115.024349)

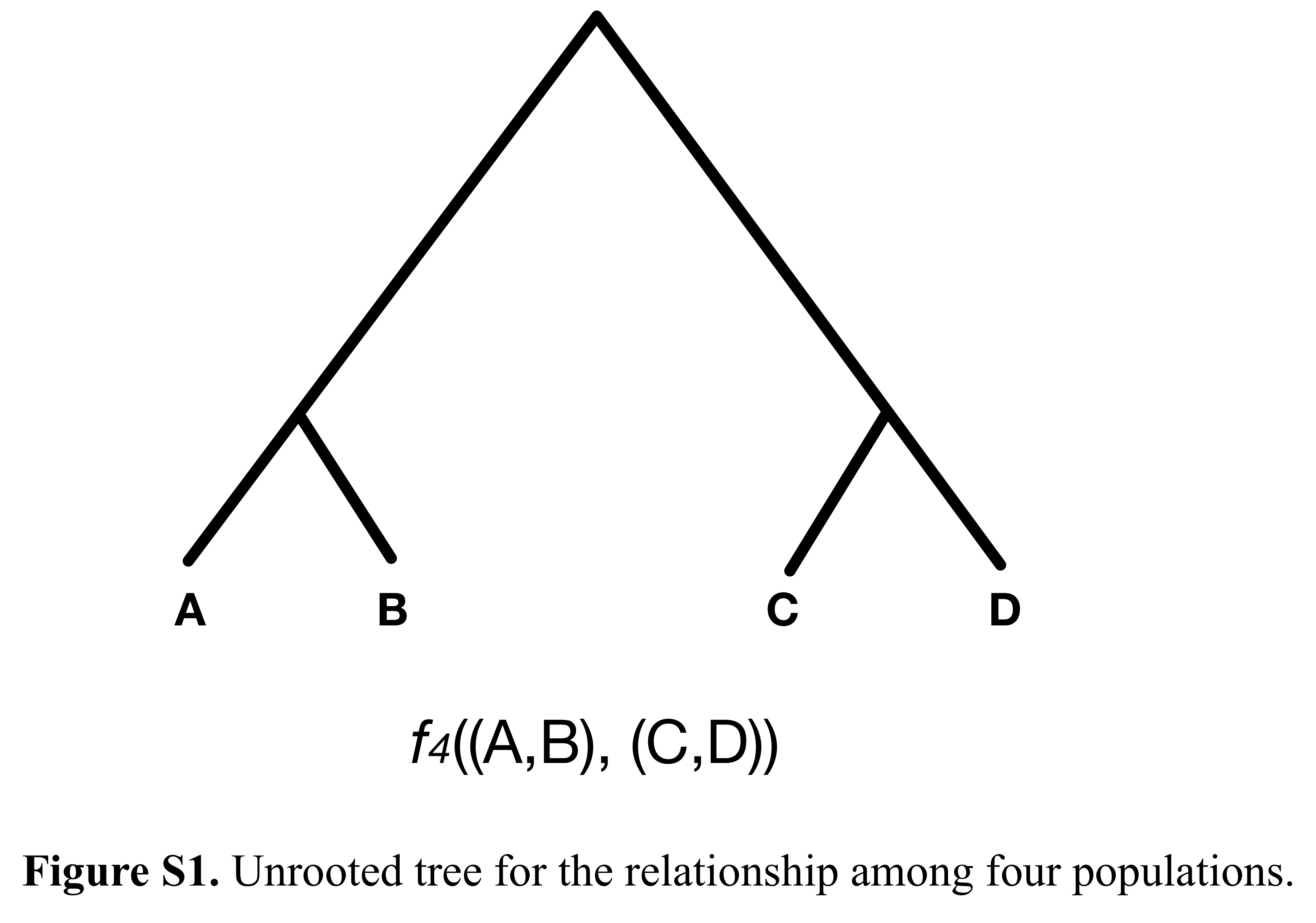

Supplement: Supporting Information [file supp_g3.115.024349_FigureS01.tif]

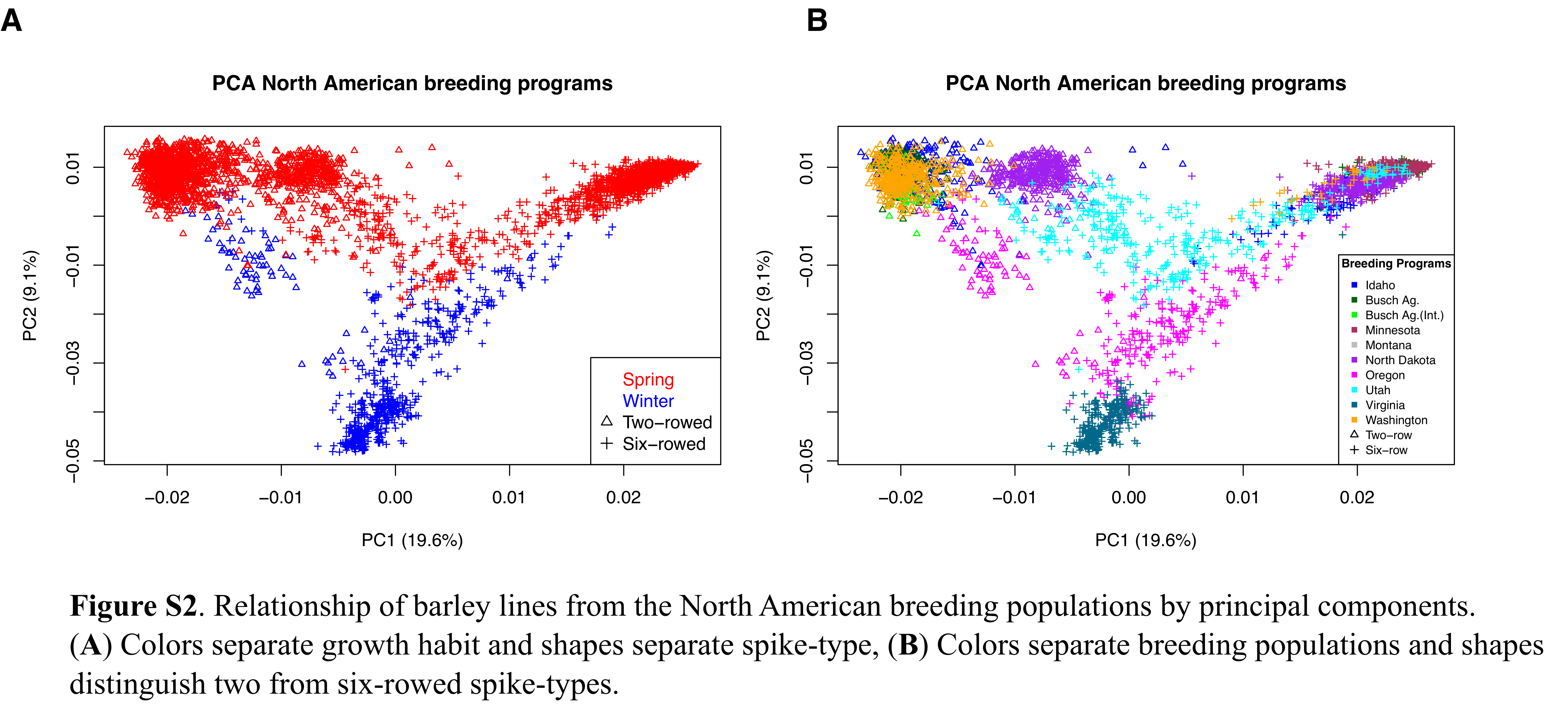

Supplement: Supporting Information [file supp_g3.115.024349_FigureS02.tif]

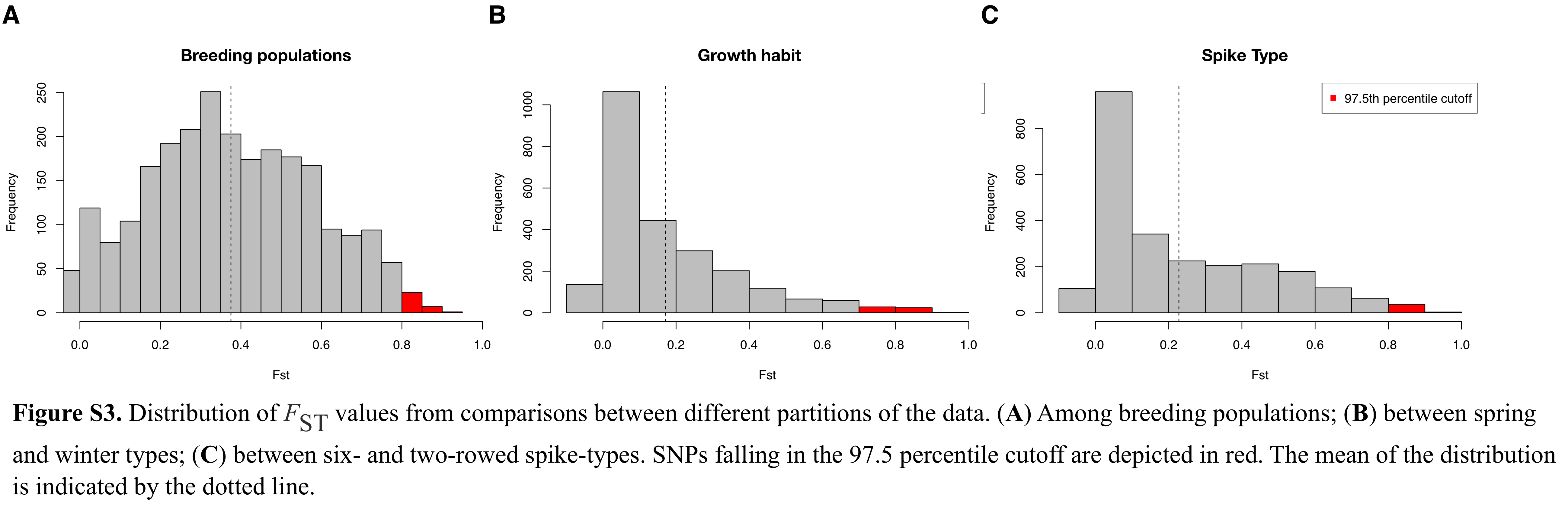

Supplement: Supporting Information [file supp_g3.115.024349_FigureS03.tif]

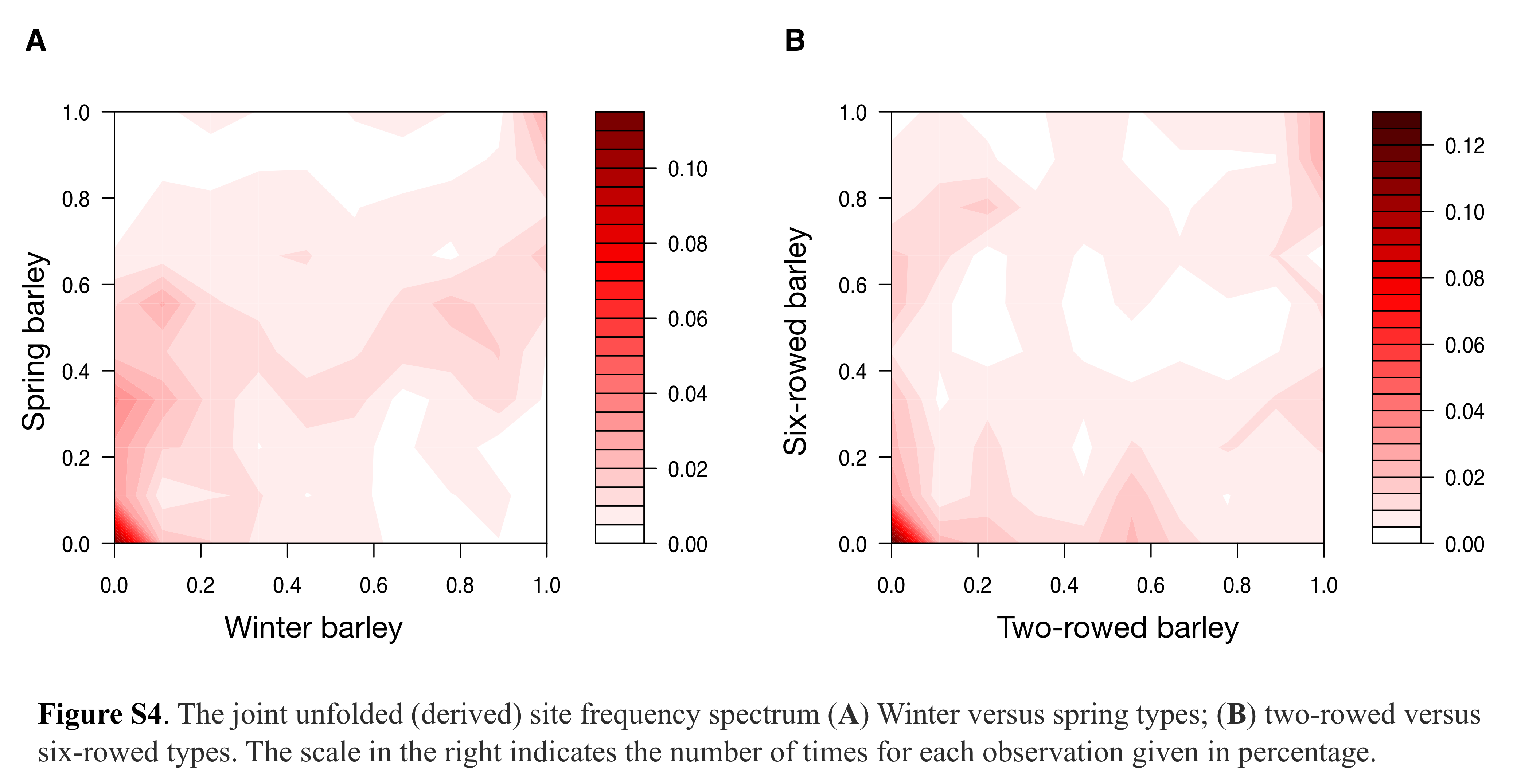

Supplement: Supporting Information [file supp_g3.115.024349_FigureS04.tif]

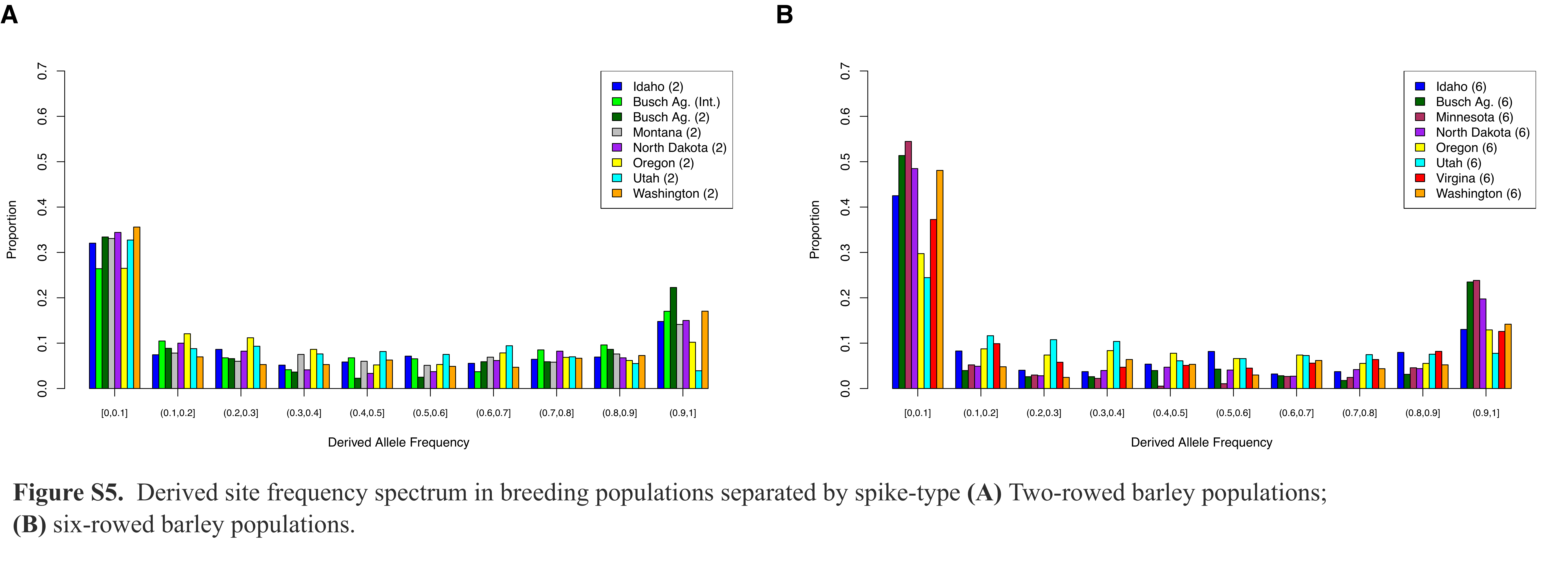

Supplement: Supporting Information [file supp_g3.115.024349_FigureS05.tif]

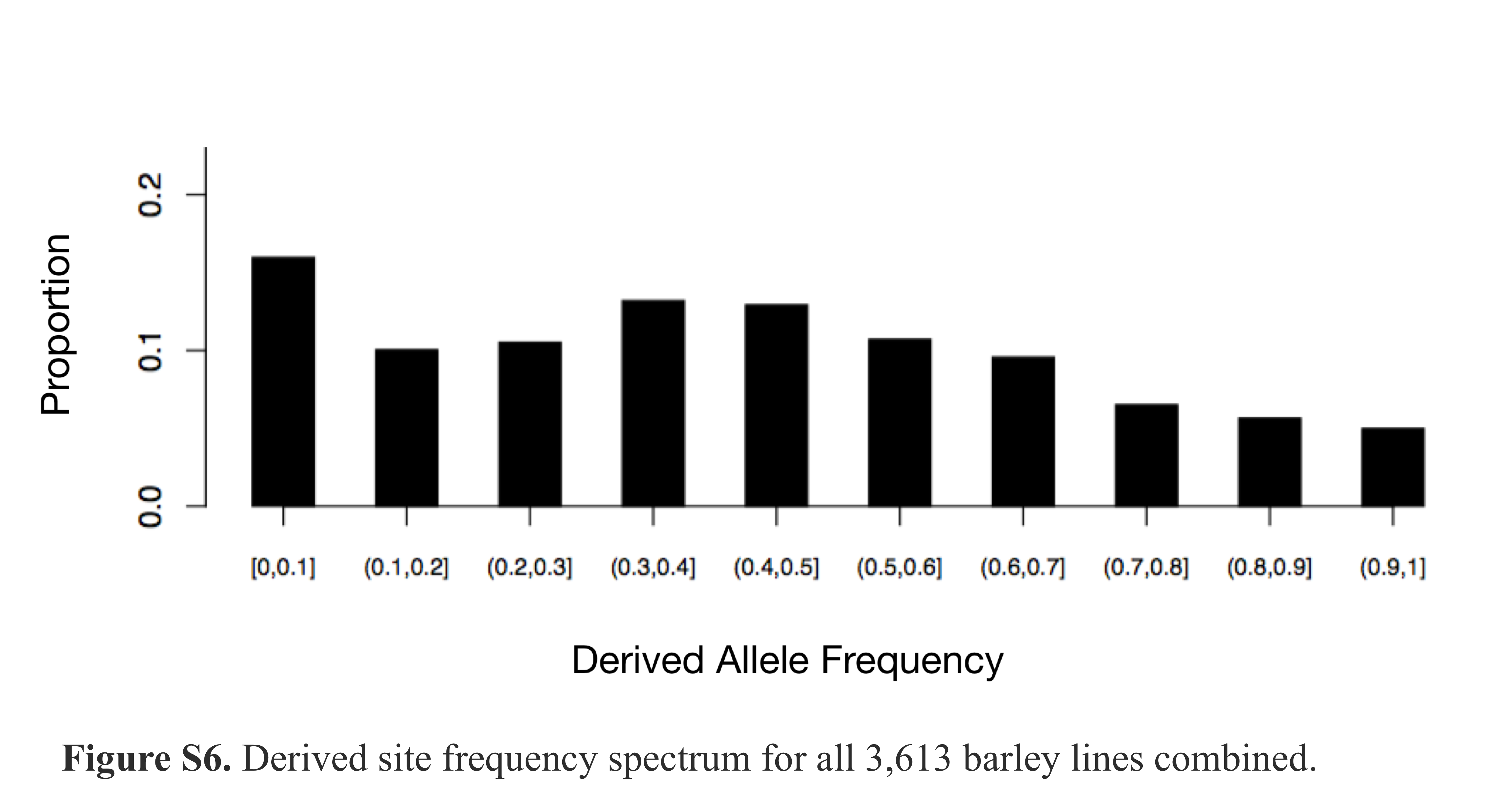

Supplement: Supporting Information [file supp_g3.115.024349_FigureS06.tif]

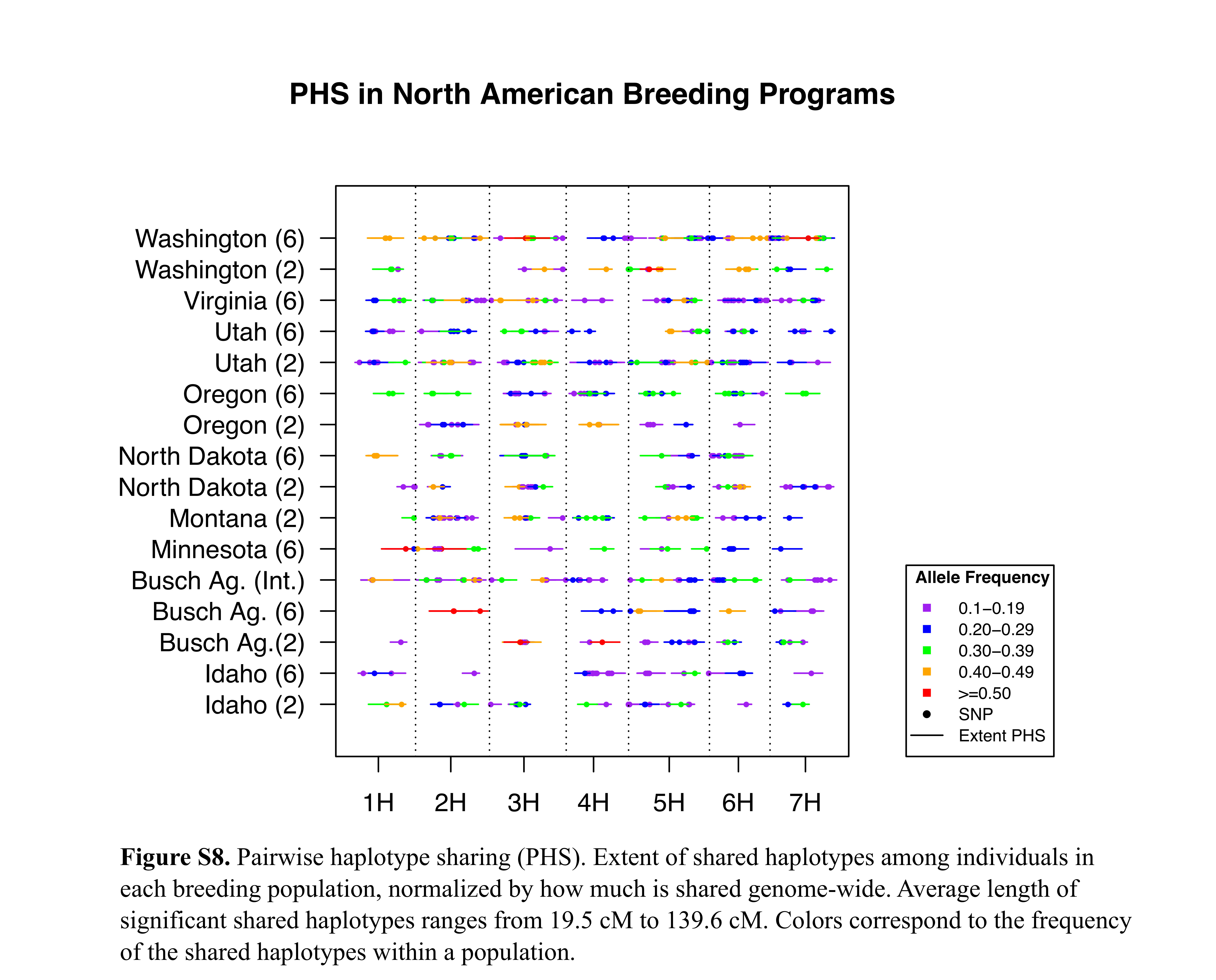

Supplement: Supporting Information [file supp_g3.115.024349_FigureS08.tif]

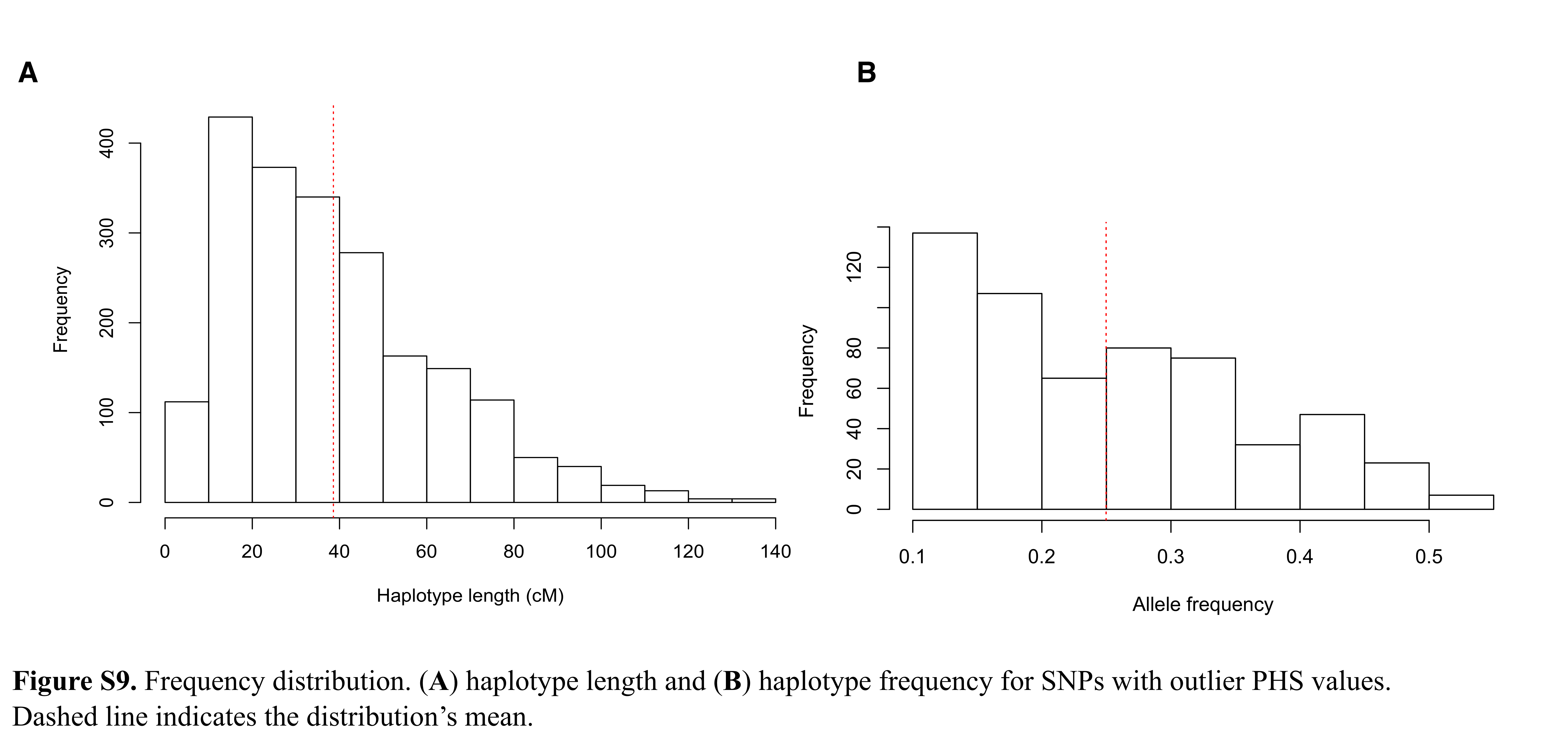

Supplement: Supporting Information [file supp_g3.115.024349_FigureS09.tif]

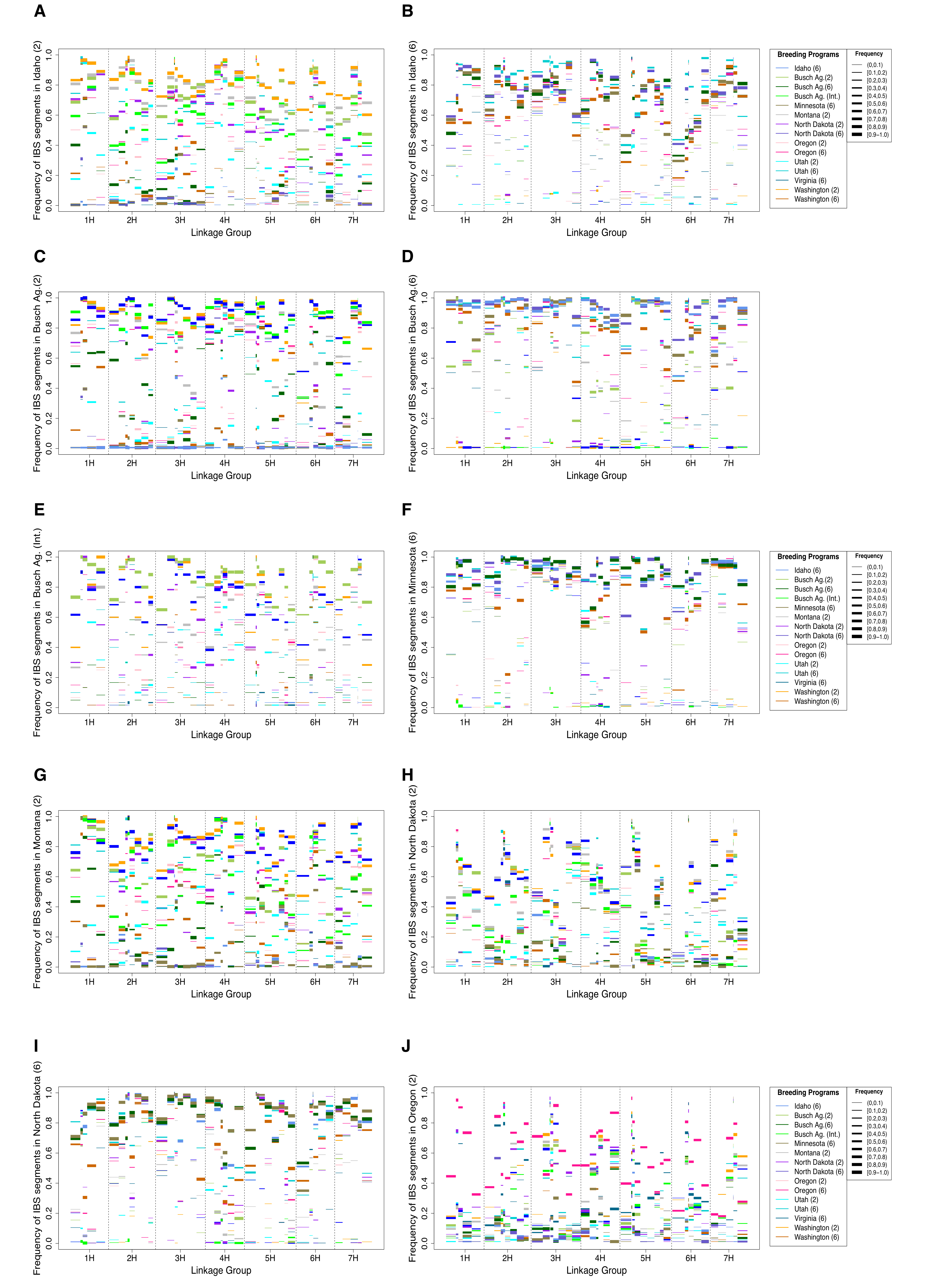

Supplement: Supporting Information [file supp_g3.115.024349_FigureS10.tif]

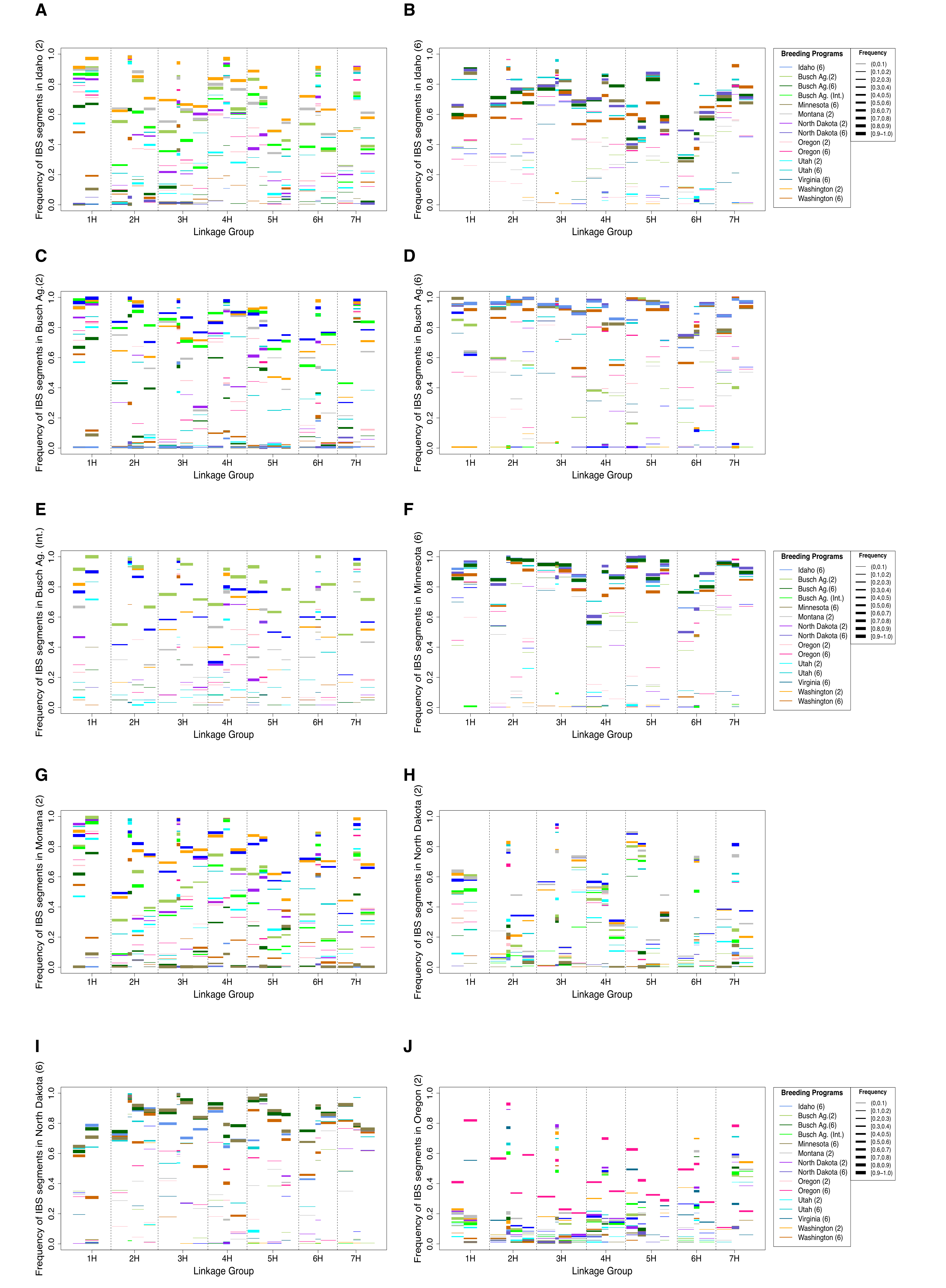

Supplement: Supporting Information [file supp_g3.115.024349_FigureS11.tif]

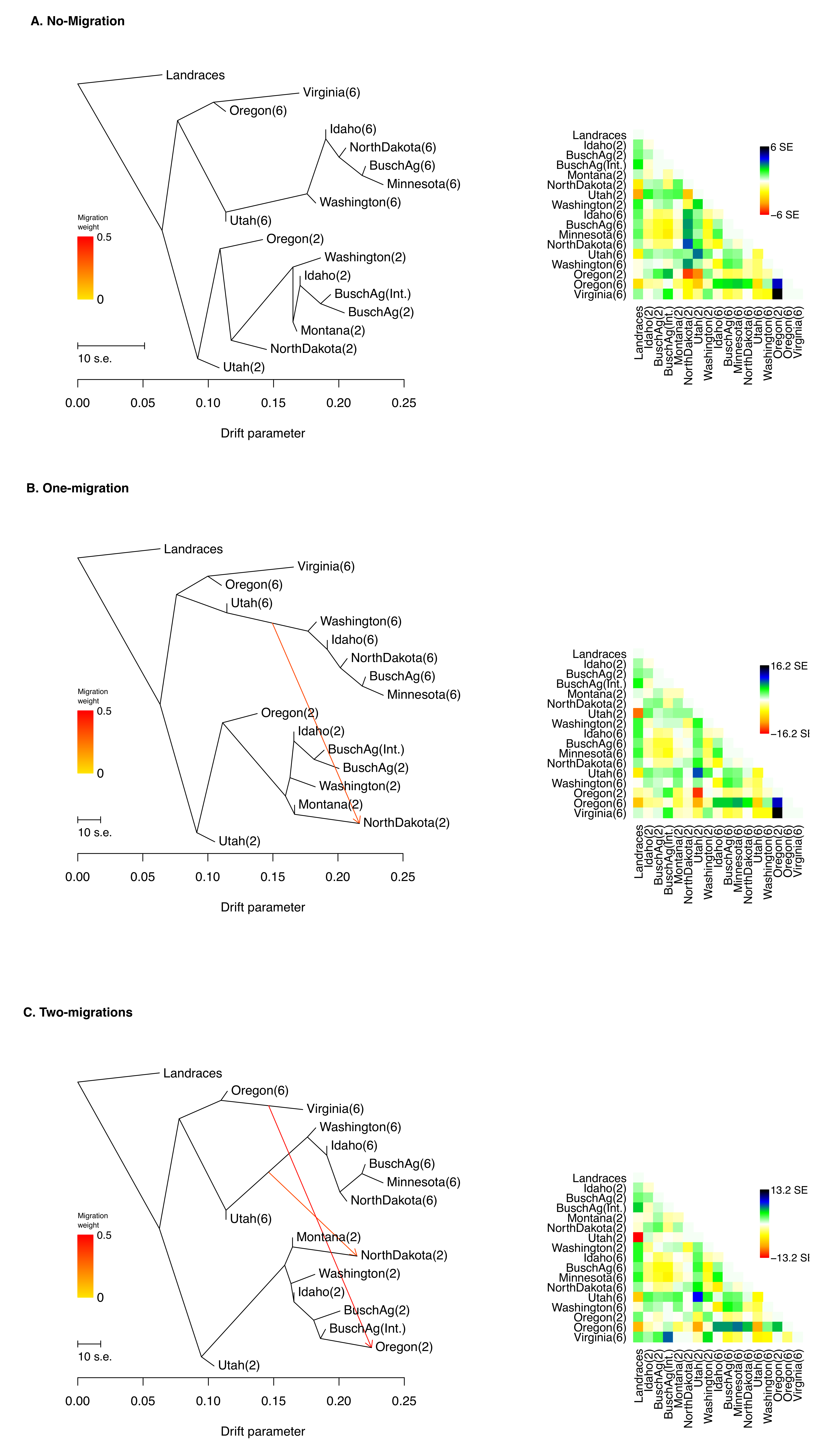

Supplement: Supporting Information [file supp_g3.115.024349_FigureS12.tif]

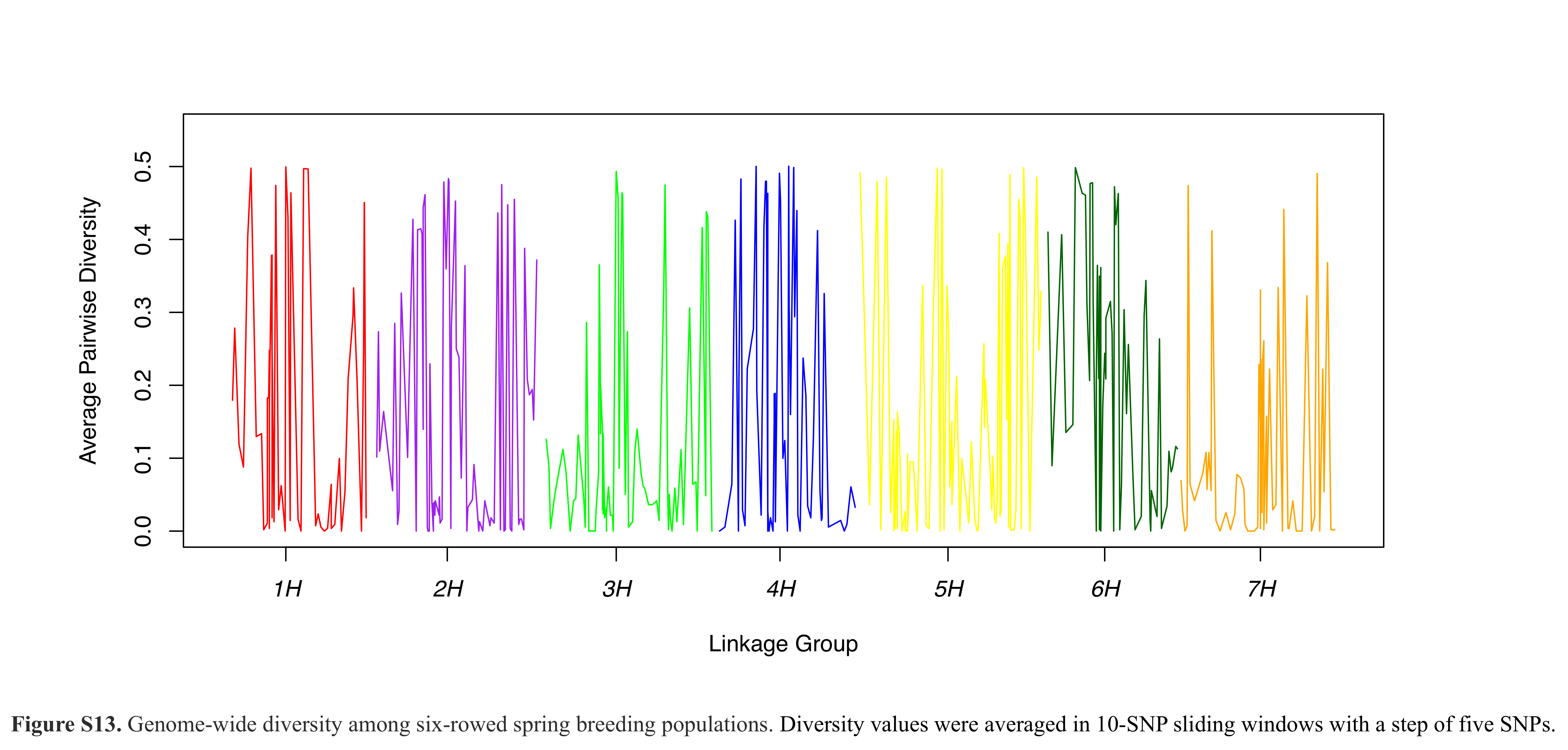

Supplement: Supporting Information [file supp_g3.115.024349_FigureS13.tif]
